# Short-Term Efficacy and Safety of IL-17, IL-12/23, and IL-23 Inhibitors Brodalumab, Secukinumab, Ixekizumab, Ustekinumab, Guselkumab, Tildrakizumab, and Risankizumab for the Treatment of Moderate to Severe Plaque Psoriasis: A Systematic Review and Network Meta-Analysis of Randomized Controlled Trials

**DOI:** 10.1155/2019/2546161

**Published:** 2019-09-10

**Authors:** Fan Bai, Gang Gang Li, Qingmin Liu, Xinwu Niu, Ruilian Li, Huiqun Ma

**Affiliations:** The Second Affiliated Hospital of Xi'an Jiaotong University, Xi'an, China

## Abstract

**Background:**

The role of interleukin-12 (IL-12), interleukin-23 (IL-23), and interleukin-17 (IL-17) has been recognized in psoriasis pathogenesis, and new drugs targeting this axis have already been developed which may provide a new therapeutic approach for patients with moderate to severe psoriasis.

**Objective:**

To compare the direct and indirect evidences of the efficacy and safety of brodalumab, secukinumab, ixekizumab, ustekinumab, guselkumab, tildrakizumab, and risankizumab in the short-term treatment of moderate to severe plaque psoriasis using network meta-analysis (NMA).

**Methods:**

A comprehensive literature search was performed in PubMed, EMBASE, and Cochrane Central Register of Controlled Trials for the available relevant studies. NMA was conducted by Stata 15.0 software using relative risks (RR) with 95% confidence interval to assess the clinical effectiveness and safety. Ranked the efficacy and safety for each drug accordance with the surface under the cumulative ranking curve (SUCRA).

**Results:**

This meta-analysis included 28 studies. All the interventions performed better than placebo in short-term achievement. Based on the result of SUCRA, ixekizumab 80 mg every 2 weeks ranked the highest in short-term achievement of PASI 75 (SUCRA = 93.0%). Brodalumab 210 mg ranked the highest in short-term achievement of PASI 100 (SUCRA = 85.0%). Secukinumab 300 mg ranked the highest in short-term achievement of sPGA 0/1 or IGA 0/1 or PGA 0/1 (SUCRA = 98.1%). In terms of having a risk of adverse events, the rates were higher in brodalumab, secukinumab, ixekizumab, and ustekinumab 45 mg compared with placebo. Ixekizumab 80 mg every 4 weeks ranked the highest in the risk of adverse events during short-term treatment (SUCRA = 4.5%). Guselkumab 50 mg ranked the highest in the risk of serious adverse events during short-term treatment (SUCRA = 25.9%). Ixekizumab 80 mg every 4 weeks ranked the highest in the risk of discontinuations due to adverse events during short-ter treatment (SUCRA = 10.7%).

**Conclusions:**

IL-17, IL-12/23, and IL-23 inhibitors had high efficacy in the achievement of PASI 75, PASI 100, and sPGA 0/1 or IGA 0/1 or PGA 0/1 in moderate to severe plaque psoriasis after 12 or 16 weeks of treatment. IL-17 inhibitors showed superior efficacy. However, its clinical safety was poor. Risankizumab appeared to have relatively high efficacy and low risk. The clinical tolerance of other biological agents needs to be further observed.

## 1. Introduction

Psoriasis is a common chronic inflammatory skin disease whose main pathological manifestations were inflammation, hyperproliferation of the epidermis, altered maturation of the epidermis, and vascular alterations [1]. The prevalence of this disease ranges from 0.51% to 11.43% in different countries [2]. Itching is the main symptom in different degrees; it has a great influence on the quality of life of patients and easily leads to social and psychological disorder such as inferiority, depression, and anxiety [3]. The pathogenesis of psoriasis is always believed to be a combination of immunologic disarrangement, psoriasis-associated susceptibility loci, psoriasis autoantigens, and multiple environmental factors; however, current research shows that psoriasis is a T-cell mediated disease primarily driven by pathogenic T-cells [4]. In an animal experiment, it is observed in the imiquimod-induced psoriasis-like mice that the epidermal expression of IL-23, IL-17A, and IL-17F is increased, whereas disease development was almost completely blocked in mice deficient for IL-23 or the IL-17 receptor [5]. Moreover, some of these studies did explore that IL-23 which is secreted by dermal dendritic cells (DDC) can induce the activation of Th17 lymphocytes and lead to the release of proinflammatory cytokines such as IL-17A, IL-17F, IL-22, IL-26, TNF-*α*, and IFN-*γ*. The complex interactions of these cytokines ultimately lead to epidermal hyperplasia, recruitment of neutrophils, and angiogenesis [6]. Recently, several studies have investigated that IL-12 and IL-23 share a common subunit (p40). Transgenic mice that overexpress IL-12 p40 develop inflammatory skin lesions [7]. Therefore, based on the theory of IL-12 and IL-23/IL-17 signal transduction pathway, blocking the important loci of signal axis has become a potential therapy to destroy the inflammatory cycle of psoriasis.

Fortunately, IL12/23, IL-17, and newer IL-23p19 antagonists have been produced and applied in clinics which shows a translational revolution in the treatment and management of psoriasis. Several studies have summarized the efficacy and safety data of IL-17 and IL-23 agents, but the results are inadequate [8, 9]. In addition, other NMA were made at the class level of medications, but not at the dosage level [10]. Therefore, we perform a systematic review with the NMA of all randomized trials to compare short-term treatment efficacy and safety of IL-17, IL-12/23, and IL-23 inhibitors brodalumab, secukinumab, ixekizumab, ustekinumab, guselkumab, tildrakizumab, and risankizumab at the dosage level for moderate to severe plaque psoriasis.

## 2. Materials and Methods

### 2.1. Study Identification

Our NMA was conducted in accordance with the Preferred Reporting Items for Systematic Reviews and Meta-analyses (PRISMA) statement [11].

A computer-based literature search was performed to identify the available relevant studies published before August 1, 2018, in PubMed, EMBASE, and Cochrane Central Register of Controlled Trials (CENTRAL), and clinical trials registered at ClinicalTrials.gov were searched for details of any relevant clinical trials in progress. We used the terms “ustekinumab or stelara or CNTO 1275 or guselkumab or tildrakizumab or SCH 900222 or MK-3222 or risankizumab or BI 655066 or secukinumab or cosentyx or AIN 457 or brodalumab or siliq or AMG-827 or lumicef or ixekizumab or taltz or LY2439821” and “psoriasis.” Vocabulary and syntax were adapted to be appropriate for each database. Standardized filters were applied for study designs, including the Cochrane highly sensitive search strategy for randomized controlled trials. Language was restricted to English. Comments, editorials, and letters were removed. The search strategy was shown in Supplementary [Supplementary-material supplementary-material-1].

### 2.2. Study Selection

We determined the inclusion and exclusion criteria before the search. The included studies should fulfill the following criteria: study design was limited to randomized, double-blind, placebo-controlled trials; adult patients (age > 18 years) are of either sex with a diagnosis of moderate to severe plaque psoriasis; the study should provide at least one efficacy outcome for short-term treatment: (1) 75% or greater reduction from baseline in the psoriasis area and severity index (PASI 75), (2) 100% reduction from baseline in the psoriasis area and severity index (PASI 100), (3) static physician's global assessment score of 0 or 1 (sPGA 0/1), (4) modified investigator's global assessment score of 0 or 1 (IGA 0/1), and (5) physician's global assessment score of 0 or 1 (PGA 0/1); the study should provide at least one safety outcome for short-term treatment: (1) one or more adverse events (AEs), (2) one or more serious adverse events (sAEs), and (3) discontinuations due to AEs. The exclusion criteria were as follows: the patients with psoriasis were under 18 years of age.

### 2.3. Data Abstraction and Quality Assessment

Two independent investigators abstracted the data using a standard data extraction form, and any disagreement will be resolved by a third author. The following information will be extracted from each included article: author, year of publication, journal, drug, time to evaluate, primary endpoint, details of the interventions, sample size, male proportion, age, duration of psoriasis, involved body surface area (%), and baseline psoriasis area and severity index (PASI) score.

Two authors independently assessed the quality of each included study in accordance with the Cochrane handbook of systematic reviews of interventions 5.1.0 (updated March 2011), which covers the following: (1) random sequence generation (selection bias), (2) allocation concealment (selection bias), (3) blinding of participants and treatment providers (performance bias), (4) blinding of outcome assessors (detection bias), (5) incomplete outcome data (attrition bias), (6) selective reporting (reporting bias), and (7) other biases. Disagreements between the review authors over the risk of bias in particular studies were resolved by a third review author.

### 2.4. Statistical Analysis

Clinical data were synthesized through narrative review with tabulation of the results of the included studies. Where sufficient clinically and statistically homogenous data were available, data were pooled using appropriate meta-analytic techniques.

Stata V.15.0 (StataCorp, College Station, TX, USA) [12] in a frequentist framework was used to perform network meta-analysis (NMA) which can make direct and indirect evidences comparable. No matter what interventions the subjects actually received, they were grouped according to the initial random statistics. If only percentages were reported, the results were estimated according to the nearest total number of events. When there was a missing value, we contacted the authors to provide the missing outcome data. Otherwise, the treatment was assumed to fail. We used the netleague command to report the RRs with corresponding 95% confidence intervals (CIs) between two comparisons, and the results were presented in a tabular form. We also ranked the efficacy and safety for each drug. The surface under the cumulative ranking curve (SUCRA) ranges from 0% to 100% were determined as an estimation of the ranking probability for each medication. For efficacy indicators, the larger the area under the curve, the better the effect. For safety indicators, the larger the area under the curve, the better the tolerance. The inconsistency and statistical disagreement between direct and indirect evidences were performed by the loop-specific method in each loop locally. And the relative odds ratio (ROR) with 95% CIs could be used to calculate clinical authenticity of NMA. If the ROR is close to 1 or the 95% CIs include 0, the similar effect estimations for direct evidence and indirect evidence are consistent [13]. Additional sensitivity analyses were performed by excluding the trials at the high risk of bias to evaluate the robustness of our findings. Publication bias was estimated by comparison-adjusted funnel plots.

## 3. Results

### 3.1. Search Strategy

**Figure 1:**
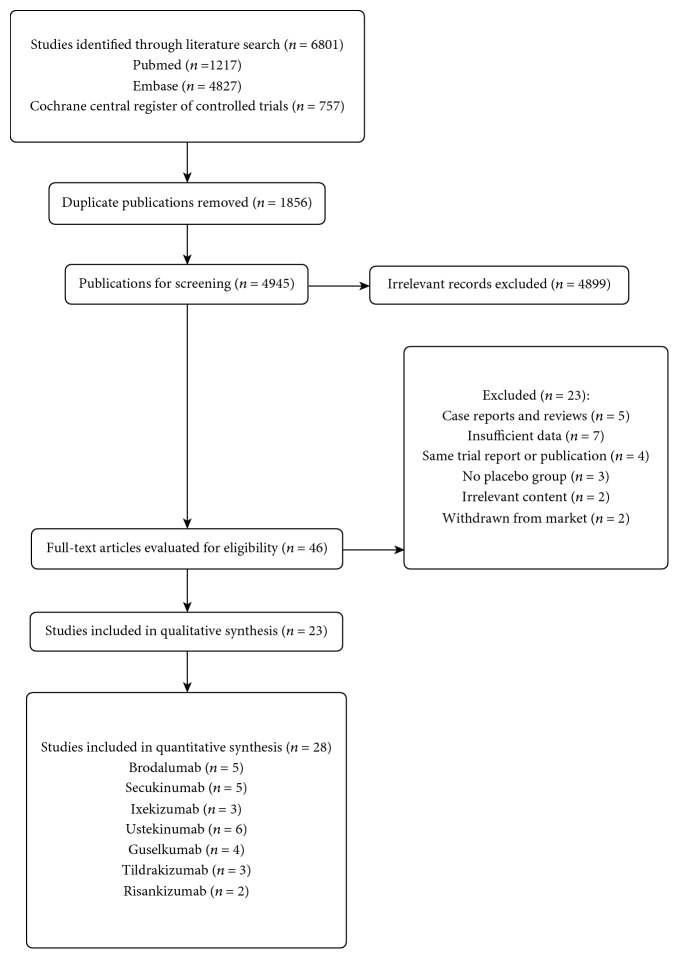
Flowchart for the selection of eligible studies.

In total, 6801 publications matching the search criteria were identified. After removing the duplicate publications, titles and abstracts of the remaining 4945 publications were screened. 4899 publications were irrelevant records, and 46 publications were ultimately determined with eligibility. Based on the inclusion and exclusion criteria, 23 articles including 28 randomized, double-blind, placebo-controlled clinical trials were assessed in this meta-analysis [14–36]: 5 trials of brodalumab, 5 trials of secukinumab, 3 trials of ixekizumab, 6 trials of ustekinumab, 4 trials of guselkumab, 3 trials of tildrakizumab, and 2 trials of risankizumab. The flowchart for the selection of eligible studies is shown in Figure 1.

The characteristics of the included studies are shown in Table 1. 19840 patients with psoriasis in 28 trials were included in this meta-analysis. Except 7 phase II trials, the rest were all phase III trials. 21 trials evaluated the short-term outcomes at weeks 12 and 7 trials at weeks 16. We collected the major clinical responses and safety indicators such as PASI 75, PASI 100, sPGA 0/1, IGA 0/1, PGA 0/1, AEs, SAEs, and discontinuations due to AEs in Table 2.

### 3.2. Risk of Bias

**Figure 2:**
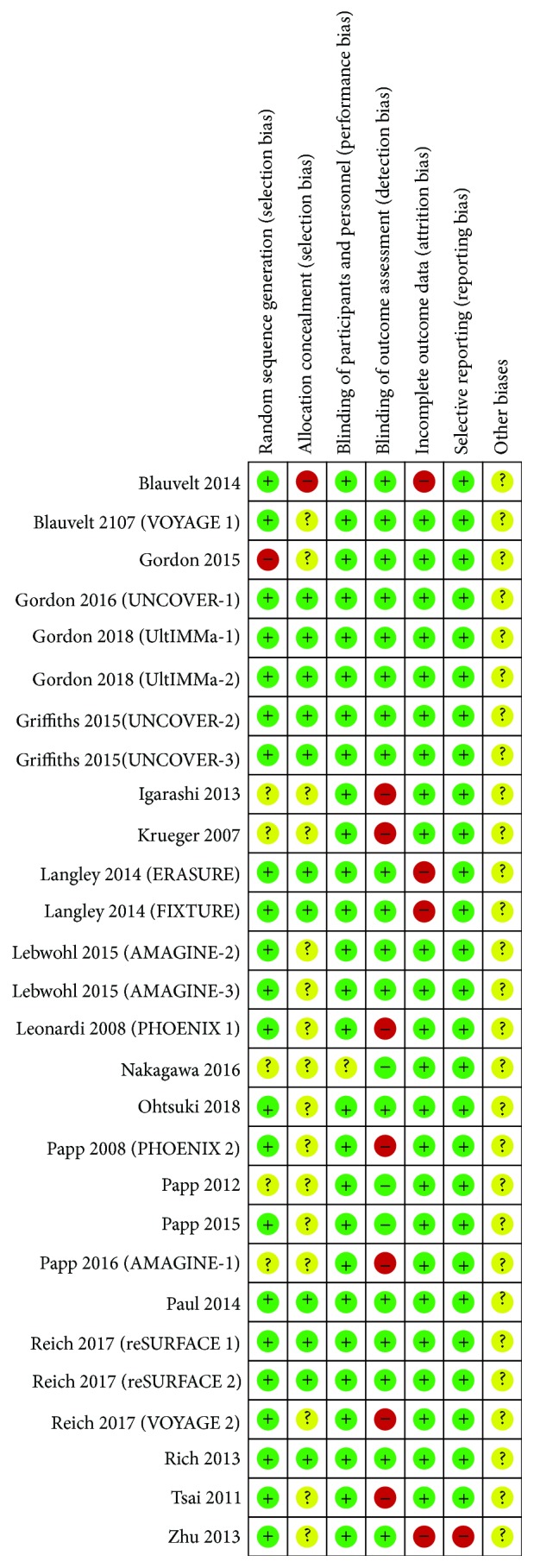
Risk of bias summary.

We summarized the risk of bias in Figure 2.

#### 3.2.1. Random Sequence Generation

Among the 28 studies, the patients in 22 studies [14–17, 19, 21–23, 25–27, 31–36] were randomized via computer-programmed random sequence or random number generator and were thus evaluated having the low risk of bias. Five studies [18, 20, 28–30] did not mention the method or detail of random sequence generation and were evaluated having an unclear risk of bias. The patients in 1 study [24] were not administered in a blinded, placebo-controlled manner evaluated as the high risk of bias.

#### 3.2.2. Allocation Concealment

11 studies [14, 16, 17, 33–36] used a validated system that automated the random assignment of medication numbers or sequentially numbered containers and were given a low risk of bias; 16 studies [18–32] did not describe any method to blind the random sequence and were evaluated having an unclear risk of bias. One study [15] divided the patients in turns and was regarded having a high risk of bias.

#### 3.2.3. Blinding of Participants and Treatment Providers

One study [29] did not specify how to perform blinding and was regarded having an unclear risk of bias; the rest of the studies were all double or triple blinded with a low risk of bias.

#### 3.2.4. Blinding of Outcome Assessors

21 studies [14–17, 23, 24, 29–36] specified that the evaluators are blinded and were given a low risk of bias. Seven studies [18–22, 25, 28] only blinded the participants and personnel and were given a high risk of bias.

#### 3.2.5. Incomplete Outcome Data

Four studies [15, 16, 23] did not report the reasons for missing data and were given a high risk of bias. The rest of the studies which performed the statistical analysis of data based on intention to treat were regarded having a low risk of bias.

#### 3.2.6. Selective Reporting

One study [23] did not preset the major outcome indicators, so we considered the quality of this study as a high risk of bias; the rest of the studies registered the protocol and reported the main outcomes and were evaluated having a low risk of bias.

#### 3.2.7. Other Biases

The evidence to judge the other biases was not enough, so we regarded the other biases of all the studies as an unclear risk of bias.

### 3.3. Network Meta-Analysis

#### 3.3.1. Network Plot

**Figure 3:**
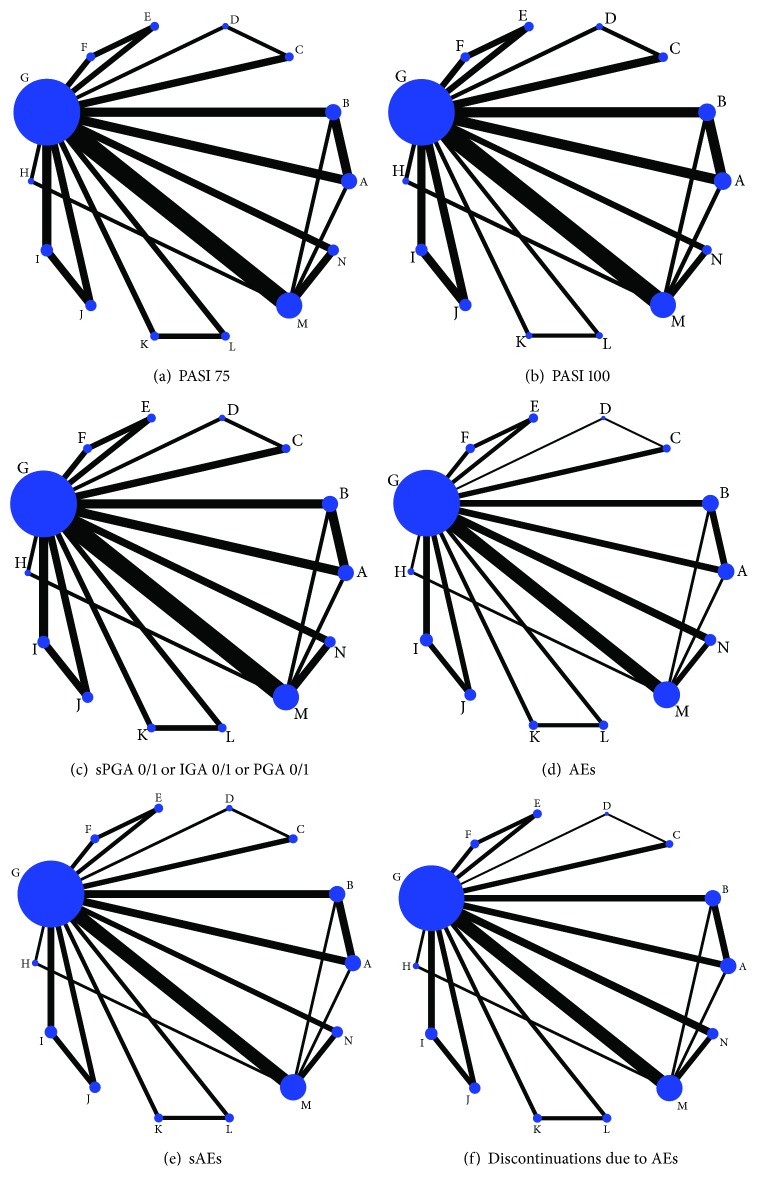
Network plots for all the evaluated outcomes at 12 or 16 weeks. The sizes of the nodes are weighted by the sample of interventions, and the widths of lines are weighed by the number of the studies involved. PASI 75: the percentages of patients with a 75% improvement from baseline in the PASI score; PASI 100: the percentages of patients with a 100% improvement from baseline in the PASI score; sPGA 0/1: static physician's global assessment score of 0 or 1; IGA 0/1: a response of 0 or 1 on the modified investigator's global assessment; PGA 0/1: physician's global assessment score of 0 or 1; AEs: adverse events; sAEs: serious adverse events. Alphabetic reference: A, brodalumab 140 mg; B, brodalumab 210 mg; C, guselkumab 100 mg; D, guselkumab 50 mg; E, ixekizumab 80 mg Q2W; F, ixekizumab 80 mg Q4W; G, placebo; H, risankizumab 150 mg; I, secukinumab 150 mg; J, secukinumab 300 mg; K, tildrakizumab 100 mg; L, tildrakizumab 200 mg; M, ustekinumab 45 mg; and N, ustekinumab 90 mg.

We built 6 networks involving 6 major outcomes. And each network plot involved 13 different dosages of 7 different biologics. The summarized network plots of the comparisons are provided in Figure 3. The number of both studies and subjects on ustekinumab 45 mg was the most frequent among all the interventions.

**Figure 4:**
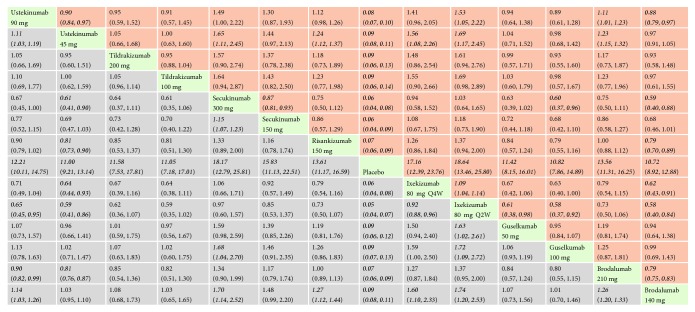
Relative risk with 95% CIs of all interventions from network meta-analysis for PASI 75. Different interventions in the middle block divide the graph into upper and lower triangles; for the lower triangle, the efficacy estimate is the ratio of the column defining treatment to the row defining treatment. In case that the confidence interval does not include 1, if RR > 1, it favors the column defining treatment. In contrast, if RR < 1, it favors the row defining treatment. The upper triangle is symmetrical to the lower triangle. The efficacy estimate is the ratio of the row defining treatment to the column defining treatment. The results are mutually reciprocal. Statistically significant results have been applied with italic formatting.

*(1) PASI 75*. With regard to PASI 75, all the interventions performed better than placebo, and the effect size was the strongest for ixekizumab 80 mg every 2 weeks (RR = 18.64, 95% CI 13.46, 25.80) and secukinumab 300 mg (RR = 18.17, 95% CI 12.79, 25.81). In the mixed comparisons, ixekizumab 80 mg every 2 weeks was superior compared with ixekizumab 80 mg every 4 weeks (RR = 1.09, 95% CI 1.04, 1.14), ustekinumab 90 mg (RR = 1.53, 95% CI 1.05, 2.22), ustekinumab 45 mg (RR = 1.69, 95% CI 1.17, 2.45), guselkumab 100 mg (RR = 1.72, 95% CI 1.09, 2.72), guselkumab 50 mg (RR = 1.63, 95% CI 1.02, 2.61), and brodalumab 140 mg (RR = 1.74, 95% CI 1.20, 2.53). Secukinumab 300 mg was more effective than secukinumab 150 mg (RR = 1.15, 95% CI 1.07, 1.23), ustekinumab 45 mg (RR = 1.65, 95% CI 1.11, 2.45), guselkumab 100 mg (RR = 1.68, 95% CI 1.04, 2.70), and brodalumab 140 mg (RR = 1.70, 95% CI 1.14, 2.52). Risankizumab 150 mg performed better than ustekinumab 45 mg (RR = 1.24, 95% CI 1.12, 1.37) and brodalumab 140 mg (RR = 1.27, 95% CI 1.12, 1.44). There was no significant difference in the efficacy of ixekizumab 80 mg every 2 weeks and secukinumab 300 mg (RR = 0.97, 95% CI 0.60, 1.57). Network meta-analysis results for PASI 75 are presented in Figure 4. The corresponding forest plot is detailed in Supplementary [Supplementary-material supplementary-material-1].

**Figure 5:**
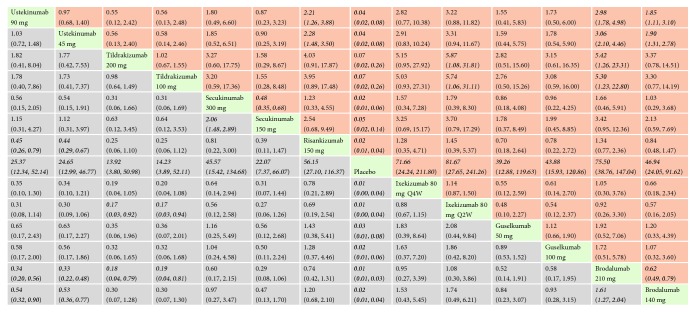
Relative risk with 95% CIs of all interventions from network meta-analysis for PASI 100. Different interventions in the middle block divide the graph into upper and lower triangles; for the lower triangle, the efficacy estimate is the ratio of the column defining treatment to the row defining treatment. In case that the confidence interval does not include 1, if RR > 1, it favors the column defining treatment. In contrast, if RR < 1, it favors the row defining treatment. The upper triangle is symmetrical to the lower triangle. The efficacy estimate is the ratio of the row defining treatment to the column defining treatment. The results are mutually reciprocal. Statistically significant results have been applied with italic formatting.

*(2) PASI 100*. With regard to PASI 100, all the interventions were significantly superior than placebo. The effect size was the strongest for ixekizumab 80 mg every 2 weeks (RR = 81.67, 95% CI 27.65, 241.26) and brodalumab 210 mg (RR = 75.50, 95% CI 38.76, 147.04). In the mixed comparisons, brodalumab 210 mg was more effective than brodalumab 140 mg (RR = 1.61, 95% CI 1.27, 2.04), ustekinumab 90 mg (RR = 2.98, 95% CI 1.78, 4.98), ustekinumab 45 mg (RR = 3.06, 95% CI 2.10, 4.46), tildrakizumab 200 mg (RR = 5.42, 95% CI 1.26, 23.31), and tildrakizumab 100 mg (RR = 5.30, 95% CI 1.23, 22.80). Brodalumab 140 mg performed better than ustekinumab 90 mg (RR = 1.85, 95% CI 1.11, 3.10) and ustekinumab 45 mg (RR = 1.90, 95% CI 1.31, 2.78). Ixekizumab 80 mg every 2 weeks was found to be more efficacious than tildrakizumab 200 mg (RR = 5.87, 95% CI 1.08, 31.81) and tildrakizumab 100 mg (RR = 5.74, 95% CI 1.06, 31.11). Network meta-analysis results for PASI 100 are presented in Figure 5. The corresponding forest plot is detailed in Supplementary [Supplementary-material supplementary-material-1].

**Figure 6:**
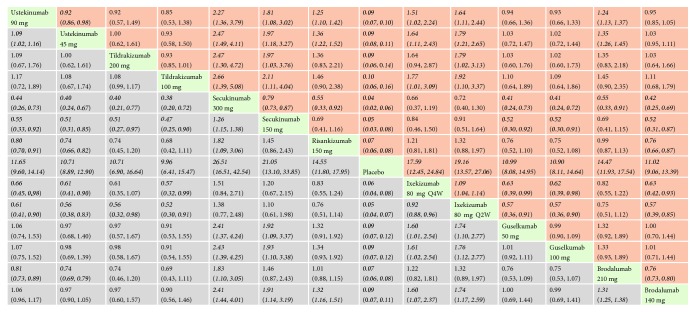
Relative risk with 95% CIs of all interventions from network meta-analysis for sPGA 0/1 or IGA 0/1 or PGA 0/1 responses. Different interventions in the middle block divide the graph into upper and lower triangles; for the lower triangle, the efficacy estimate is the ratio of the column defining treatment to the row defining treatment. In case that the confidence interval does not include 1, if RR > 1, it favors the column defining treatment. In contrast, if RR < 1, it favors the row defining treatment. The upper triangle is symmetrical to the lower triangle. The efficacy estimate is the ratio of the row defining treatment to the column defining treatment. The results are mutually reciprocal. Statistically significant results have been applied with italic formatting.

*(3) sPGA 0/1 or IGA 0/1 or PGA 0/1*. With regard to sPGA 0/1 or IGA 0/1 or PGA 0/1, the biologics involved were statistically significantly superior to placebo. The effect size was the strongest for secukinumab 300 mg (RR = 26.51, 95% CI 16.51, 42.54) and secukinumab 150 mg (RR = 21.05, 95% CI 13.10, 33.85). In the mixed comparisons, secukinumab 300 mg was more effective than secukinumab 150 mg (RR = 1.26, 95% CI 1.15, 1.38), ustekinumab 90 mg (RR = 2.27, 95% CI 1.36, 3.79), tildrakizumab 200 mg (RR = 2.47, 95% CI 1.30, 4.72), risankizumab 150 mg (RR = 1.82, 95% CI 1.09, 3.06), guselkumab 100 mg (RR = 2.43, 95% CI 1.39, 4.25), and brodalumab 210 mg (RR = 1.83, 95% CI 1.10, 3.05). Brodalumab 210 mg performed better than ustekinumab 90 mg (RR = 1.24, 95% CI 1.13, 1.37) and ustekinumab 45 mg (RR = 1.35, 95% CI 1.26, 1.45). Network meta-analysis results for sPGA 0/1 or IGA 0/1 or PGA 0/1 responses are presented in Figure 6. The corresponding forest plot is detailed in Supplementary [Supplementary-material supplementary-material-1].

**Figure 7:**
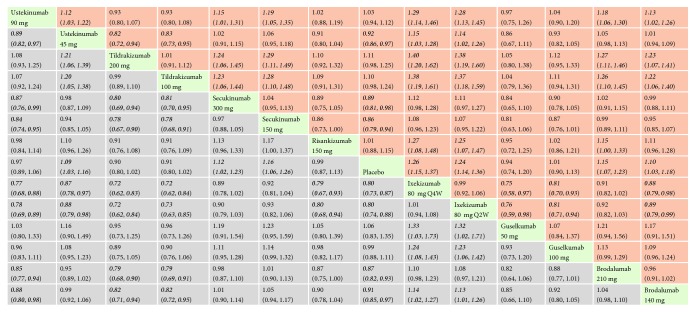
Relative risk with 95% CIs of all interventions from network meta-analysis for adverse events(AEs). Different interventions in the middle block divide the graph into upper and lower triangles; for the lower triangle, the efficacy estimate is the ratio of the column defining treatment to the row defining treatment. In case that the confidence interval does not include 1, if RR > 1, it favors the row defining treatment. In contrast, if RR < 1, it favors the column defining treatment. The upper triangle is symmetrical to the lower triangle. The efficacy estimate is the ratio of the row defining treatment to the column defining treatment. The results are mutually reciprocal. Statistically significant results have been applied with italic formatting.

*(4) AEs*. In terms of having a risk of AEs, the rate was higher in secukinumab 300 mg, secukinumab 150 mg, ustekinumab 45 mg, brodalumab 210 mg and brodalumab 140 mg, ixekizumab 80 mg every 4 weeks, and ixekizumab 80 mg every 2 weeks compared to placebo. The effect size was the strongest for ixekizumab 80 mg every 4 weeks (RR = 1.26, 95% CI 1.15, 1.37) and ixekizumab 80 mg every 2 weeks (RR = 1.24, 95% CI 1.14, 1.36). In the mixed comparisons, secukinumab 150 mg was more likely to result in AEs than ustekinumab 90 mg (RR = 1.19, 95% CI 1.05, 1.35), tildrakizumab 200 mg (RR = 1.29, 95% CI 1.11, 1.49), and tildrakizumab 100 mg (RR = 1.28, 95% CI 1.10, 1.48). There was no significant difference between ixekizumab 80 mg every 2 weeks and ixekizumab 80 mg every 4 weeks (RR = 1.01, 95% CI 0.94, 1.08). Network meta-analysis results for AEs are presented in Figure 7. The corresponding forest plot is detailed in Supplementary [Supplementary-material supplementary-material-1].

**Figure 8:**
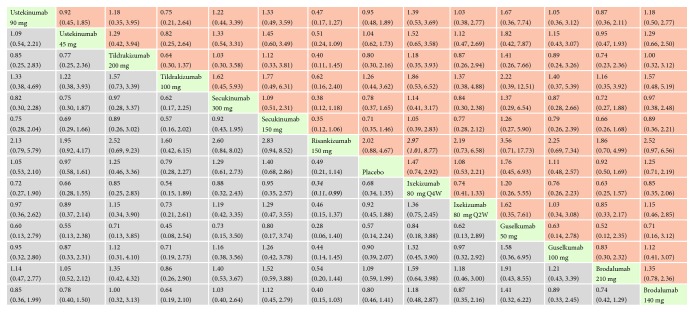
Relative risk with 95% CIs of all interventions from network meta-analysis for serious adverse events (sAEs). Different interventions in the middle block divide the graph into upper and lower triangles; for the lower triangle, the efficacy estimate is the ratio of the column defining treatment to the row defining treatment. In case that the confidence interval does not include 1, if RR > 1, it favors the row defining treatment. In contrast, if RR < 1, it favors the column defining treatment. The upper triangle is symmetrical to the lower triangle. The efficacy estimate is the ratio of the row defining treatment to the column defining treatment. The results are mutually reciprocal. Statistically significant results have been applied with italic formatting.

*(5) sAEs*. In terms of having a risk of sAEs, no significant difference was observed between these biologics and placebo. Besides, in the mixed comparisons, the rate was higher in ixekizumab 80 mg every 4 weeks compared to risankizumab 150 mg (RR = 2.97, 95% CI 1.01, 8.77). Network meta-analysis results for sAEs are presented in Figure 8. The corresponding forest plot is detailed in Supplementary [Supplementary-material supplementary-material-1].

**Figure 9:**
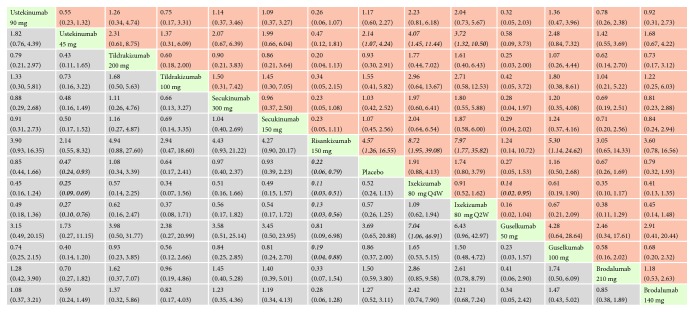
Relative risk with 95% CIs of all interventions from network meta-analysis for Discontinuations due to AEs. Different interventions in the middle block divide the graph into upper and lower triangles; for the lower triangle, the efficacy estimate is the ratio of the column defining treatment to the row defining treatment. In case that the confidence interval does not include 1, if RR > 1, it favors the row defining treatment. In contrast, if RR < 1, it favors the column defining treatment. The upper triangle is symmetrical to the lower triangle. The efficacy estimate is the ratio of the row defining treatment to the column defining treatment. The results are mutually reciprocal. Statistically significant results have been applied with italic formatting.

*(6) Discontinuations due to AEs*. In terms of having a risk of discontinuations due to AEs, ustekinumab 45 mg and risankizumab 150 mg present a relatively lower risk than placebo; RR were 0.47 (95% CI 0.24, 0.93) and 0.22 (95% CI 0.06, 0.79), respectively. In the mixed comparisons, ixekizumab 80 mg every 4 weeks was more likely to result in discontinuations due to AEs than ustekinumab 45 mg (RR = 4.07, 95% CI 1.45, 11.44), risankizumab 150 mg (RR = 8.72, 95% CI 1.95, 39.08), and guselkumab 50 mg (RR = 7.04, 95% CI 1.06, 46.91). Network meta-analysis results for discontinuations are presented in Figure 9. The corresponding forest plot is detailed in Supplementary [Supplementary-material supplementary-material-1].

### 3.4. Ranking of Treatments by Efficacy

**Figure 10:**
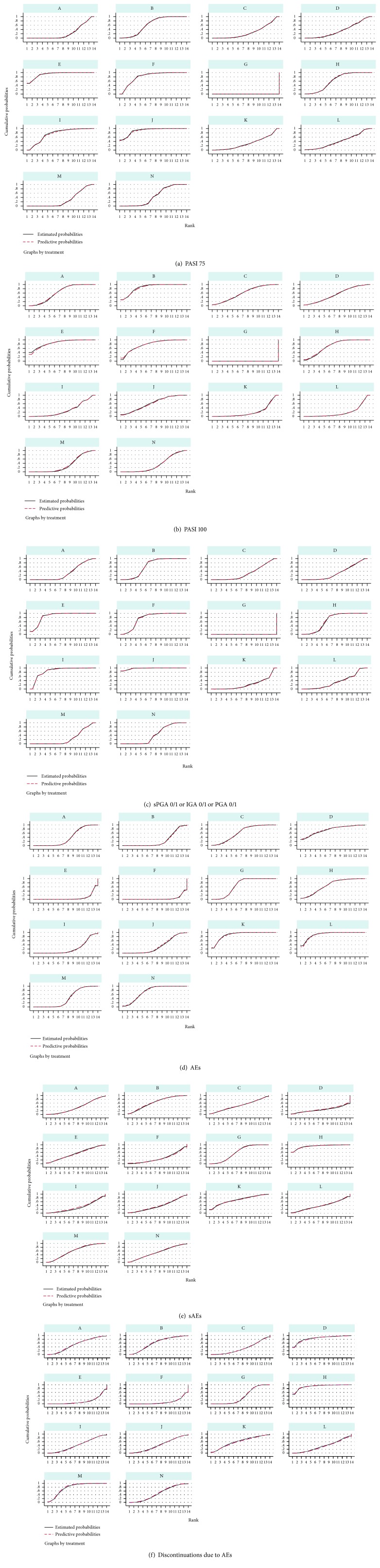
Surface under the cumulative ranking curves (SUCRA) for all interventions of all outcomes in the network meta-analysis. PASI 75: the percentages of patients with a 75% improvement from baseline in the PASI score; PASI 100: the percentages of patients with a 100% improvement from baseline in the PASI score; sPGA 0/1: static physician's global assessment score of 0 or 1; IGA 0/1: a response of 0 or 1 on the modified investigator's global assessment; PGA 0/1: physician's global assessment score of 0 or 1; AEs: adverse events; sAEs: serious adverse events. Alphabetic reference: A, brodalumab 140 mg; B, brodalumab 210 mg, C, guselkumab 100 mg; D, guselkumab 50 mg; E, ixekizumab 80 mg Q2W; F, ixekizumab 80 mg Q4W; G, placebo; H, risankizumab 150 mg; I, secukinumab 150 mg; J, secukinumab 300 mg; K, tildrakizumab 100 mg; L, tildrakizumab 200 mg; M, ustekinumab 45 mg; and N, ustekinumab 90 mg.

The summarized efficacy and safety ranking of the 13 interventions according to their surface under the cumulative ranking curves (SUCRA) are shown in Table 3; SUCRA for each treatment included in the network are presented in Figure 10. The ranking for short-term achievements of PASI 75 from high to low was as follows: ixekizumab 80 mg every 2 weeks (SUCRA: 93.0%), secukinumab 300 mg (SUCRA: 89.9%), ixekizumab 80 mg every 4 weeks (SUCRA: 81.5%), secukinumab 150 mg (SUCRA: 73.5%), brodalumab 210 mg (SUCRA: 62.5%), risankizumab 150 mg (SUCRA: 62.3%), ustekinumab 90 mg (SUCRA: 44.5%), tildrakizumab 200 mg (SUCRA: 42.2%), guselkumab 50 mg (SUCRA: 38.7%), tildrakizumab 100 mg (SUCRA: 33.2%), guselkumab 100 mg (SUCRA: 28.5%), ustekinumab 45 mg (SUCRA: 27.4%), and brodalumab 140 mg (SUCRA: 22.8%). The ranking for short-term achievements of PASI 100 from high to low was as follows: brodalumab 210 mg (SUCRA: 85.0%), ixekizumab 80 mg every 2 weeks (SUCRA: 83.3%), ixekizumab 80 mg every 4 weeks (SUCRA: 76.8%), risankizumab 150 mg (SUCRA: 71.3%), brodalumab 140 mg (SUCRA: 63.4%), secukinumab 300 mg (SUCRA: 62.4%), guselkumab 100 mg (SUCRA: 61.4%), guselkumab 50 mg (SUCRA: 55.9%), ustekinumab 90 mg (SUCRA: 34.5%), ustekinumab 45 mg (SUCRA: 33.1%), secukinumab 150 mg (SUCRA: 31.0%), tildrakizumab 100 mg (SUCRA: 21.9%), and tildrakizumab 200 mg (SUCRA: 20.0%). The ranking for short-term achievements of sPGA 0/1 or IGA 0/1 or PGA 0/1 from high to low was as follows: secukinumab 300 mg (SUCRA: 98.1%), ixekizumab 80 mg every 2 weeks (SUCRA: 86.5%), secukinumab 150 mg (SUCRA: 85.7%), ixekizumab 80 mg every 4 weeks (SUCRA: 75.7%), risankizumab 150 mg (SUCRA: 66.7%), brodalumab 210 mg (SUCRA: 65.4%), ustekinumab 90 mg (SUCRA: 42.8%), tildrakizumab 200 mg (SUCRA: 34.7%), guselkumab 50 mg (SUCRA: 33.4%), brodalumab 140 mg (SUCRA: 32.0%), guselkumab 100 mg (SUCRA: 31.4%), ustekinumab 45 mg (SUCRA: 25.4%), and tildrakizumab 100 mg (SUCRA: 22.3%).

### 3.5. Ranking of Treatments by Safety

According to the SUCRA, the ranking for the short-term risk of adverse events from high to low was as follows: ixekizumab 80 mg every 4 weeks (SUCRA: 4.5%), ixekizumab 80 mg every 2 weeks (SUCRA: 7.5%), secukinumab 150 mg (SUCRA: 22.7%), brodalumab 210 mg (SUCRA: 23.7%), secukinumab 300 mg (SUCRA: 33.9%), brodalumab 140 mg (SUCRA: 38.1%), ustekinumab 45 mg (SUCRA: 41.0%), guselkumab 100 mg (SUCRA: 63.4%), risankizumab 150 mg (SUCRA: 67.6%), ustekinumab 90 mg (SUCRA: 75.2%), guselkumab 50 mg (SUCRA: 76.2%), tildrakizumab 100 mg (SUCRA: 88.8%), and tildrakizumab 200 mg (SUCRA: 90.2%). The ranking for the short-term risk of serious adverse events from high to low was as follows: guselkumab 50 mg (SUCRA: 25.9%), ixekizumab 80 mg every 4 weeks (SUCRA: 27.5%), secukinumab 150 mg (SUCRA: 30.7%), secukinumab 300 mg (SUCRA: 35.6%), brodalumab 140 mg (SUCRA: 38.4%), tildrakizumab 200 mg (SUCRA: 41.1%), guselkumab 100 mg (SUCRA: 49.4%), ixekizumab 80 mg every 2 weeks (SUCRA: 50.8%), ustekinumab 90 mg (SUCRA: 52.5%), ustekinumab 45 mg (SUCRA: 61.1%), brodalumab 210 mg (SUCRA: 63.9%), tildrakizumab 100 mg (SUCRA: 70.8%), and risankizumab 150 mg (SUCRA: 92.8%). The ranking for the short-term risk of discontinuations due to adverse events from high to low was as follows: ixekizumab 80 mg every 4 weeks (SUCRA: 10.7%), ixekizumab 80 mg every 2 weeks (SUCRA: 14.8%), guselkumab 100 mg (SUCRA: 32.1%), tildrakizumab 200 mg (SUCRA: 35.4%), secukinumab 300 mg (SUCRA: 42.2%), secukinumab 150 mg(SUCRA: 43.5%), ustekinumab 90 mg (SUCRA: 49.7%), brodalumab 140 mg (SUCRA: 54.2%), tildrakizumab 100 mg (SUCRA: 58.6%), brodalumab 210 mg (SUCRA: 63.0%), ustekinumab 45 mg (SUCRA: 79.0%), guselkumab 50 mg (SUCRA: 84.6%), and risankizumab 150 mg (SUCRA: 92.6%).

### 3.6. Inconsistency

**Figure 11:**
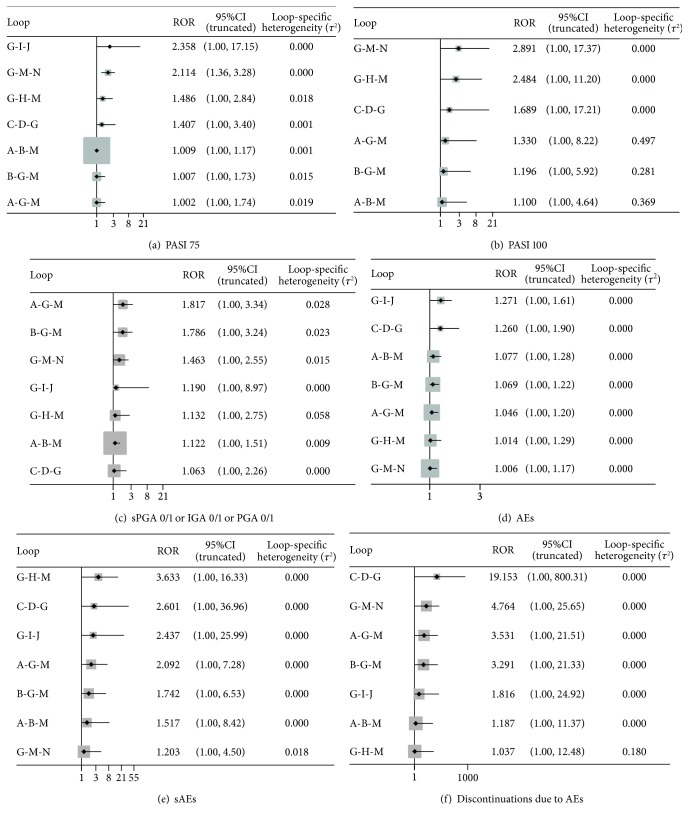
Inconsistency in closed loops for all the outcomes. The graph shows the estimates of differences between direct and indirect comparisons as represented by the relative odds ratio (ROR) with 95% CIs. PASI 75: the percentages of patients with a 75% improvement from baseline in the PASI score; PASI 100: the percentages of patients with a 100% improvement from baseline in the PASI score; sPGA 0/1: static physician's global assessment score of 0 or 1; IGA 0/1: a response of 0 or 1 on the modified investigator's global assessment; PGA 0/1: physician's global assessment score of 0 or 1; AEs: adverse events; sAEs: serious adverse events. Alphabetic reference: A, brodalumab 140 mg; B, brodalumab 210 mg; C, guselkumab 100 mg; D, guselkumab 50 mg; E, ixekizumab 80 mg Q2W; F, ixekizumab 80 mg Q4W; G, placebo; H, risankizumab 150 mg; I, secukinumab 150 mg; J, secukinumab 300 mg; K, tildrakizumab 100 mg; L, tildrakizumab 200 mg; M, ustekinumab 45 mg; and N, ustekinumab 90 mg.

Inconsistency refers to the difference between direct and indirect evidences, which will affect the authenticity of network meta-analysis. We used the relative odds ratio (ROR) with 95% CIs to calculate the absolute difference between direct and indirect evidences. If the ROR is close to 1, or the 95% CIs include 0, the effect estimations for direct and indirect evidences are consistent. The results of PASI 100, sPGA 0/1 or IGA 0/1 or PGA 0/1, AEs, sAEs, and discontinuations due to AEs showed no significant inconsistencies in all closed loops which revealed the consistency model's conclusions were robust. For PASI 75, there was statistical loop inconsistency in the loop containing placebo, ustekinumab 45 mg, and ustekinumab 90 mg in the combined results of direct and indirect evidences (ROR = 2.114, 95% CI 1.36, 3.28). Inconsistency plots in closed loops for all the outcomes are shown in Figure 11.

### 3.7. Sensitivity Analysis

Considered PASI 100 and sPGA 0/1 or IGA 0/1 or PGA 0/1 responses are more relevant to the psoriasis patient, we performed sensitivity analyses with these outcomes by excluding the trials at a high risk of bias to evaluate the robustness of our findings. Results were consistent with the main analysis for the efficacy outcomes. And the forest plots are detailed in Supplementary [Supplementary-material supplementary-material-1]-[Supplementary-material supplementary-material-1].

#### 3.7.1. Publication Bias

**Figure 12:**
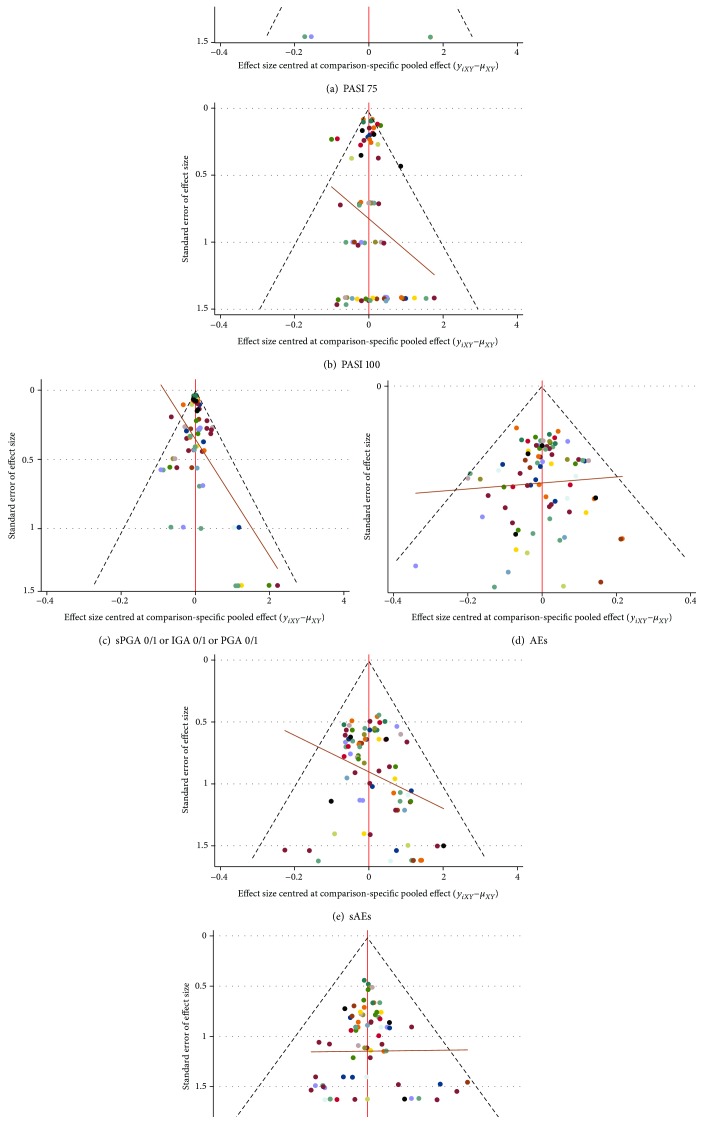
Comparison-adjusted funnel plots of all the outcomes in network meta-analysis. PASI 75: the percentages of patients with a 75% improvement from baseline in the PASI score; PASI 100: the percentages of patients with a 100% improvement from baseline in the PASI score; sPGA 0/1: static physician's global assessment score of 0 or 1; IGA 0/1: a response of 0 or 1 on the modified investigator's global assessment; PGA 0/1: physician's global assessment score of 0 or 1; AEs: adverse events; sAEs: serious adverse events. Alphabetic reference: A, brodalumab 140 mg; B, brodalumab 210 mg; C, guselkumab 100 mg; D, guselkumab 50 mg; E, ixekizumab 80 mg Q2W; F, ixekizumab 80 mg Q4W; G, placebo; H, risankizumab 150 mg; I, secukinumab 150 mg; J, secukinumab 300 mg; K, tildrakizumab 100 mg; L, tildrakizumab 200 mg; M, ustekinumab 45 mg; and N, ustekinumab 90 mg.

Comparison-adjusted funnel plots of all the outcomes in network meta-analysis are shown in Figure 12. We found no evidence of publication bias in the result of “AEs” and “discontinuations due to AEs.” However, the results of other outcomes were not absolutely symmetrical which suggested publication bias may exist.

## 4. Discussion

In recent years, biological agents have been widely used in dermatology, especially in patients with chronic psoriasis. Many clinical trials have shown that biological agents can quickly control the illness and improve life quality. Meanwhile, the safety of biological agents has also attracted the attention of dermatologists. Therefore, we summarized and evaluated the short-term therapeutic efficacy and safety of IL-17, IL-12/23, and IL-23 biological agents for the treatment of moderate to severe plaque psoriasis. We extracted direct and indirect evidences from 28 trials with 19840 patients into this meta-analysis. Among these studies, except for 7 phase II trials [17, 18, 20, 24, 29, 30, 32], the others were all phase III trials. All of them were placebo-controlled, and a parallel assignment study contained at least two different doses with the exception of 2 trials that included only one dose with no active controller [19, 23]. In addition, the primary endpoints were all assessed at 12 or 16 weeks.

In order to make the efficacy and safety data of these medications comparable, we performed a NMA to compare the indirect evidences in this quantitative meta-analysis. We found that all biologics involved were more efficacious than placebo in achieving PASI 75, PASI 100, and sPGA 0/1 or IGA 0/1 or PGA 0/1 responses. IL-17 inhibitors achieved outstanding performance in the treatment effect at 12 weeks compared with IL-12/23 and IL-23 inhibitors. For PASI 75, ixekizumab 80 mg every 2 weeks ranked first, followed by secukinumab 300 mg, ixekizumab 80 mg every 4 weeks, secukinumab 150 mg and brodalumab 210 mg. For PASI 100, brodalumab 210 mg ranked first, followed by ixekizumab 80 mg every 2 weeks and ixekizumab 80 mg every 4 weeks. Studies showed that about 42% of patients treated with brodalumab 210 mg reached PASI 100 at 12 weeks, while only 0.4% of patients treated with placebo reached PASI 100 at 12 weeks [28]. For sPGA 0/1 or IGA 0/1 or PGA 0/1 responses, secukinumab 300 mg ranked first, followed by ixekizumab 80 mg every 2 weeks, secukinumab 150 mg, ixekizumab 80 mg every 4 weeks and risankizumab 150 mg. In addition, three trials of ixekizumab observed the improvement of the Dermatological Life Quality Index (DLQI) in patients with moderate to severe plaque psoriasis. Data showed that DLQI improved rapidly in the second week after the treatment with ixekizumab, and more than 60% of patients received DLQI (0/1) at 12 weeks without psoriasis symptoms [35, 36]. It was noteworthy that in the use of brodalumab, secukinumab, and ixekizumab, high-dose drugs or high-frequency drugs were more effective than low-dose drugs or low-frequency drugs. Risankizumab is a new type of IL-23 inhibitor, whose short-term efficacy was better than other IL-23 or IL-12/23 inhibitors. The results for short-term safety assessment showed that the risk of adverse events in brodalumab 210 mg, brodalumab 140 mg, secukinumab 300 mg, secukinumab 150 mg, ixekizumab 80 mg every 4 weeks, ixekizumab 80 mg every 2 weeks, and ustekinumab 45 mg was higher than that in the placebo group at 12 or 16 weeks. It showed that IL-17 inhibitors were less tolerant than other biological agents. It was also worth noting that the tolerance of ixekizumab 80 mg every 4 weeks was often worse than that of ixekizumab 80 mg every 2 weeks and was associated with greater likelihood of causing sAEs and discontinuations. There was no significant difference in the short-term adverse event risk between other biological agents and placebo. According to the clinical trials, the most relevant adverse events were nasopharyngitis, upper respiratory tract inflammation, and injection site reaction. Guselkumab is a kind of biological therapy that selectively blocked IL-23 [37]. In the 100 mg treatment group, the short-term risk of adverse events was lower than that of other biological agents; however, the risk of discontinuations was high, which may limit its clinical use. In the 50 mg treatment group, the short-term risk of adverse events and discontinuations was low, but the risk of serious adverse events ranked first, so a long-term follow-up of guselkumab is necessary to examine its treatment tolerability. The present meta-analysis included a kind of IL-12/23 inhibitor named ustekinumab which blocked not only the IL-12 but also the IL-23 [38]. Though the risk of adverse events was higher in ustekinumab 45 mg than that in placebo, the risk of discontinuations in the ustekinumab 45 mg and risankizumab 150 mg groups was lower than that in placebo. In ustekinumab 45 mg, only 6 out of 1013 patients abandoned treatment because of adverse events, while 23 out of 983 patients in the placebo group. In the risankizumab 150 mg, 3 out of 598 patients abandoned treatment because of adverse events, while 5 out of 200 patients in the placebo group [18–23, 34]. Not only that, risankizumab 150 mg had a lower risk of adverse events and serious adverse events compared with other IL-23 inhibitors, showing relatively high clinical efficacy and low treatment risk.

Our findings indicated efficacy and safety differences among the biologic agents used in moderate to severe plaque psoriasis which could provide valuable references to clinicians. The strengths in, firstly, our research included the latest biological agents for psoriasis treatment. Tildrakizumab had been licensed for treating psoriasis patients by the FDA in March 2018, and risankizumab had been approved by the FDA in Apirl 2019. Secondly, our study included a large number of patients and high quality of RCTs availabled at present. We compared different drugs at the dose level which not only increased the credibility of our research but also provided more information to the clinicians. Thirdly, previous studies took more attention on PASI 75 and PASI 90. However, PASI 100 is more relevant to the patients. In order to better reflect the efficacy of these drugs, we included PASI 100 in the evaluation of the primary outcomes.

Some potential limitations could affect the interpretation of our findings. Firstly, a placebo-controlled study was conducted during the induction period; however, the study design of the maintenance stage varied and usually the lack of a placebo-control group which increased the difficulty to extract and analyze all the data. So we evaluated the primary endpoints at the end of the induction period (12-16 weeks), whether the biologic agents can ultimately improve the quality of life of psoriasis patients is still unclear and requires more researches to continue. Secondly, some studies lack details of randomization sequence generation, allocation concealment, and blinding, which could reduce the reliability of our results. Therefore, to ensure the authenticity of the results, we made a sensitivity analysis with these outcomes by excluding the trials at a high risk of bias to evaluate the robustness of our findings. Results were consistent with the main analysis for the efficacy outcomes which could strengthen our analysis. Thirdly, few head to head trials available in our NMA, and most of the analyses were based on the indirect comparisons which may limit the consistency evaluation. Fourthly, there were more Americans and Canadians involved in the trials than Asians, so the results of this analysis may not be generalized to all the people in the world. Finally, the analysis has not considered the different medical histories of the patients, which may affect the findings and results.

## 5. Conclusions

IL-17, IL-12/23, and IL-23 inhibitors had high efficacy in the achievement of PASI 75, PASI 100, and sPGA 0/1 or IGA 0/1 or PGA 0/1 in moderate to severe plaque psoriasis after 12 or 16 weeks of treatment. IL-17 inhibitors brodalumab, secukinumab, and ixekizumab showed superior efficacy in current clinical trials. However, the clinical safety of IL-17 inhibitors was weaker than that of IL-12/23 and IL-23 inhibitors. Ustekinumab was the only IL-12/23 inhibitor included in this study which performed mediocre in both efficacy and tolerance. The IL-23 inhibitor risankizumab is an excellent performer with high efficacy and low risk. The clinical tolerance of other biological agents needs to be further observed. These results may provide a new choice for a clinical treatment of plaque psoriasis. However, further clinical head to head trials are needed to confirm the long-term efficacy and safety of action of the interventions. And treatment decisions should also be based on the associated cost-effectiveness.

## Figures and Tables

**Table 1:** Characteristics of the included studies.

Author	Year	Journal	Drug	Time to evaluate	Primary endpoint	Group	Total	Male, *N* (%)	Age (mean age)	Duration of psoriasis (years)	Involved body surface area (%)	Baseline PASI score
Papp (AMAGINE-1)	2016	BJD	Brodalumab	12	PASI 75	210 mg	222	161 (73)	46 ± 12	20 ± 13	25.1 ± 15.3	19.4 ± 6.6
					sPGA 0/1	140 mg	219	162 (74)	46 ± 13	19 ± 13	27.4 ± 17.1	20.0 ± 7.4
						Placebo	220	161 (73)	47 ± 13	21 ± 12	26.9 ± 17.1	19.7 ± 7.7

Lebwohl (AMAGINE-2)	2015	NEJM	Brodalumab	12	PASI 75	210 mg	612	421 (69)	45 ± 13	19 ± 12	26 ± 16	20.3 ± 8.3
					sPGA 0/1	140 mg	610	413 (68)	45 ± 13	19 ± 12	27 ± 17	20.5 ± 8.2
					PASI 100	Ustekinumab	300	205 (68)	45 ± 13	19 ± 13	27 ± 19	20.0 ± 8.4
						Placebo	309	219 (71)	44 ± 13	18 ± 12	28 ± 17	20.4 ± 8.2

Lebwohl (AMAGINE-3)	2015	NEJM	Brodalumab	12	PASI 75	210 mg	624	431 (69)	45 ± 13	18 ± 12	28 ± 18	20.4 ± 8.3
					sPGA 0/1	140 mg	629	437 (70)	45 ± 13	18 ± 12	29 ± 18	20.1 ± 8.5
					PASI 100	Ustekinumab	313	212 (68)	45 ± 13	17 ± 12	28 ± 18	20.1 ± 8.4
						Placebo	315	208 (66)	44 ± 13	18 ± 12	28 ± 17	20.1 ± 8.7

Papp	2012	NEJM	Brodalumab	12	PASI 75	210 mg	39	28 (72)	44.0 ± 11.7	19.2 ± 9.7	24.9 ± 16.9	19.4 ± 8.0
					PASI 100	140 mg	39	22 (56)	42.1 ± 11.1	20.7 ± 11.8	24.1 ± 12.8	18.8 ± 5.7
						Placebo	42	30 (71)	42.3 ± 12.2	19.3 ± 12.4	21.3 ± 11.0	17.9 ± 5.5

Nakagawa	2015	JDS	Brodalumab	12	PASI 75	210 mg	37	29 (78.4)	46.4 ± 11.8	15.0 ± 11.0	43.7 ± 26.0	28.0 ± 14
					PASI 100	140 mg	37	30 (81.1)	46.4 ± 13.2	14.5 ± 9.5	42.7 ± 21.2	28.5 ± 11
					sPGA 0/1	Placebo	38	27 (71.1)	46.6 ± 10.8	17.0 ± 11.4	38.0 ± 21.5	24.0 ± 9

Rich	2012	BJD	Secukinumab	12	PASI 75	150 mg	133	105 (78.9)	44.5 ± 12.5	17.4 ± 11.8	22.8 ± 14.7	19.9 ± 7.8
						Placebo	67	44 (65.7)	44.2 ± 13.0	15.4 ± 10.7	21.7 ± 16.0	20.5 ± 9.3

Paul (JUNCTURE)	2015	JEADV	Secukinumab	12	PASI 75	300 mg	60	46 (76.7)	46.6 ± 14.2	21.0 ± 13.5	26.4 ± 12.8	18.9 ± 6.4
					IGA 0/1	150 mg	61	41 (67.2)	43.9 ± 14.4	20.6 ± 14.5	30.1 ± 16.7	22.0 ± 8.9
						Placebo	61	38 (62.3)	43.7 ± 12.7	19.9 ± 12.2	25.7 ± 14.7	19.4 ± 6.7

Langley (ERASURE)	2014	NEJM	Secukinumab	12	PASI 75	300 mg	245	169 (69.0)	44.9 ± 13.5	17.4 ± 11.1	32.8 ± 19.3	22.5 ± 9.2
					IGA 0/1	150 mg	245	168 (68.6)	44.9 ± 13.3	17.5 ± 12.0	33.3 ± 19.2	22.3 ± 9.8
						Placebo	248	172 (69.4)	45.4 ± 12.6	17.3 ± 12.4	29.7 ± 15.9	21.4 ± 9.1

Langley (FIXTURE)	2014	NEJM	Secukinumab	12	PASI 75	300 mg	327	224 (68.5)	44.5 ± 13.2	15.8 ± 12.3	34.3 ± 19.2	23.9 ± 9.9
					IGA 0/1	150 mg	327	236 (72.2)	45.4 ± 12.9	17.3 ± 12.2	34.5 ± 19.4	23.7 ± 10.5
						Placebo	326	237 (72.7)	44.1 ± 12.6	16.6 ± 11.6	35.2 ± 19.1	24.1 ± 10.5

Blauvelt (FEATURE)	2015	BJD	Secukinumab	12	PASI 75	300 mg	59	38 (64.4)	45.1 ± 12.6	18.0 ± 11.9	33.3 ± 18.0	20.7 ± 8.0
					IGA 0/1	150 mg	59	40 (67.8)	46.0 ± 15.1	20.4 ± 13.0	30.6 ± 16.6	20.5 ± 8.3
						Placebo	59	39 (66.1)	46.5 ± 14.1	20.2 ± 14.2	32.2 ± 17.4	21.1 ± 8.5

Gordon (UNCOVER-1)	2016	NEJM	Ixekizumab	12	PASI 75	80 mg Q4W	432	289 (66.9)	46 ± 13	19 ± 12	27 ± 16	20 ± 8
					sPGA 0/1	80 mg Q2W	433	291 (67.2)	45 ± 12	20 ± 12	28 ± 18	20 ± 7
						Placebo	431	303 (70.3)	46 ± 13	20 ± 12	27 ± 18	20 ± 9

Griffiths (UNCOVER-2)	2015	Lancet	Ixekizumab	12	PASI 75	80 mg Q4W	347	244 (70.3)	45 ± 14	19 ± 13	27 ± 17	20 ± 7
					sPGA 0/1	80 mg Q2W	351	221 (63.0)	45 ± 13	18 ± 12	25 ± 16	19 ± 7
						Placebo	168	120 (71.4)	45 ± 12	19 ± 13	27 ± 18	21 ± 8

Griffiths (UNCOVER-3)	2015	Lancet	Ixekizumab	12	PASI 75	80 mg Q4W	386	258 (66.8)	46 ± 13	18 ± 12	28 ± 16	21 ± 8
					sPGA 0/1	80 mg Q2W	385	254 (66.0)	46 ± 13	18 ± 12	28 ± 17	21 ± 8
						Placebo	193	137 (71.0)	46 ± 12	18 ± 13	29 ± 17	21 ± 8

IGARASHI	2012	JD	Ustekinumab	12	PASI 75	45 mg	64	53 (82.8)	45	15.8 ± 8.2	47 ± 23.7	30 ± 12.9
						90 mg	62	47 (75.8)	44	17.3 ± 10.7	47 ± 19.7	29 ± 11.2
						Placebo	32	26 (83.9)	49	16.0 ± 11.2	50 ± 22.5	30 ± 11.8

Leonardi (PHOENIX 1)	2008	Lancet	Ustekinumab	12	PASI 75	45 mg	255	175 (68·6)	44.8	19.7	27.2	20.5
						90 mg	256	173 (67·6)	46.2	19.6	25.2	19.7
						Placebo	255	183 (71·8)	44.8	20.4	27.7	20.4

Papp (PHOENIX 2)	2008	Lancet	Ustekinumab	12	PASI 75	45 mg	409	283 (69·2)	45.1	19.3	25.9	19.4
						90 mg	411	274 (66·7)	46.6	20.3	27.1	20.1
						Placebo	410	283 (69·0)	47.0	20.8	26.1	19.4

Tsai (PEARL)	2011	JDS	Ustekinumab	12	PASI 75	45 mg	61	50 (82.0)	40.9 ± 12.7	11.9 ± 7.5	41.8 ± 24.4	25.2 ± 11.9
						Placebo	60	53 (88.3)	40.4 ± 10.1	13.9 ± 7.3	35.8 ± 21.4	22.9 ± 8.6

Zhu (LOTUS)	2013	JDD	Ustekinumab	12	PASI 75	45 mg	160	125 (78.1)	40.1 ± 12.4	14.6 ± 8.9	35.1 ± 18.5	23.2 ± 9.5
						Placebo	162	123 (75.9)	39.2 ± 12.2	14.2 ± 8.6	35.1 ± 19.6	22.7 ± 9.5

Krueger	2007	NEJM	Ustekinumab	12	PASI 75	45 mg	64	39 (61)	45 ± 12	19.8 ± 11.9	27.4 ± 16.9	18.9 ± 7.0
						90 mg	64	52 (81)	44 ± 13	17.3 ± 13.5	27.4 ± 18.1	19.0 ± 7.9
						Placebo	64	46 (72)	44 ± 14	16.9 ± 11.0	26.6 ± 18.4	19.9 ± 8.3

Blauvelt (VOYAGE 1)	2017	JAAD	Guselkumab	16	IGA 0/1	100 mg	329	240 (72.9)	43.9 ± 12.7	17.9 ± 12.3	28.3 ± 17.1	22.1 ± 9.5
					PASI 90	Placebo	174	119 (68.4)	44.9 ± 12.9	17.6 ± 12.4	25.8 ± 15.9	20.4 ± 8.7

Reich (VOYAGE 2)	2017	JAAD	Guselkumab	16	IGA 0/1	100 mg	496	349 (70.4)	43.7 ± 12.2	17.9 ± 12.0	28.5 ± 16.4	21.9 ± 8.8
					PASI 90	Placebo	248	173 (69.8)	43.3 ± 12.4	17.9 ± 11.9	28.0 ± 16.5	21.5 ± 8.0

Gordon	2015	NEJM	Guselkumab	16	PGA 0/1	50 mg	42	28 (67)	43	19	25	19
						100 mg	42	31 (74)	42	18	24	21
						Placebo	42	28 (67)	46.5	18	28	22

OHTSUKI	2017	JD	Guselkumab	16	IGA 0/1	50 mg	65	44 (67.7)	50.1	15.25	38.0	25.60
					PASI 90	100 mg	63	47 (74.6)	47.8	14.39	37.9	26.73
						Placebo	64	54 (84.4)	48.3	13.66	33.6	25.92

Papp	2015	BJD	Tildrakizumab	16	PASI 75	100 mg	89	76 (85)	45.5 ± 12.8	NR	NR	NR
						200 mg	86	65 (76)	43.2 ± 12.6	NR	NR	NR
						Placebo	46	38 (83)	45.9 ± 11.7	NR	NR	NR

Reich (reSURFACE 1)	2017	Lancet	Tildrakizumab	12	PASI 75	100 mg	309	207 (67)	46.4	NR	29.7	20.0
					PGA 0/1	200 mg	308	226 (73)	46.9	NR	30.9	20.7
						Placebo	155	100 (65)	47.9	NR	29.6	19.3

Reich (reSURFACE 2)	2017	Lancet	Tildrakizumab	12	PASI 75	100 mg	307	220 (72)	44.6	NR	34.2	20.5
					PGA 0/1	200 mg	314	225 (72)	44.6	NR	31.8	19.8
						Placebo	313	222 (71)	45.8	NR	31.6	20.2

Gordon (UltIMMa-1)	2018	Lancet	Risankizumab	16	PASI 90	150 mg	304	212 (70)	48.3	NR	26.2	20.6
					sPGA 0/1	Ustekinumab	100	70 (70)	46.5	NR	25.2	20.1
						Placebo	102	79 (77)	49.3	NR	27.9	20.5

Gordon (UltIMMa-1)	2018	Lancet	Risankizumab	16	PASI 90	150 mg	294	203 (69)	46.2	NR	26.2	20.5
					sPGA 0/1	Ustekinumab	99	66 (67)	48.6	NR	20.9	18.2
						Placebo	98	67 (68)	46.3	NR	23.9	18.9

Data are presented as the numbers (%) or the means ± standard deviations. PASI 75: the percentages of patients with a 75% improvement from baseline in the PASI score; PASI 90: the percentages of patients with a 90% improvement from baseline in the PASI score; PASI 100: the percentages of patients with a 100% improvement from baseline in the PASI score; sPGA 0/1: static physician's global assessment score of 0 or 1; IGA 0/1: a response of 0 or 1 on the modified investigator's global assessment; PGA 0/1: physician's global assessment score of 0 or 1; Q2W: every 2 weeks; Q4W: every 4 weeks; NR: not reported.

**Table 2:** Summary of pooled major clinical responses and adverse events during short-time treatment.

Author	Drug	Time to evaluate	Group	Total	PASI 75	PASI 100	sPGA 0/1	IGA 0/1	PGA 0/1	AEs	sAEs	Discontinuations due to AEs
Papp (AMAGINE-1)	Brodalumab	12	210 mg	222	185	93	168	NR	NR	131	4	2
			140 mg	219	132	51	118	NR	NR	126	6	3
			Placebo	220	6	1	3	NR	NR	112	3	3

Lebwohl (AMAGINE-2)	Brodalumab	12	210 mg	612	528	272	481	NR	NR	354	6	3
			140 mg	610	406	157	354	NR	NR	365	13	4
			Ustekinumab	300	210	65	183	NR	NR	177	4	2
			Placebo	309	25	2	12	NR	NR	165	8	0

Lebwohl (AMAGINE-3)	Brodalumab	12	210 mg	624	531	229	497	NR	NR	353	9	4
			140 mg	629	435	170	377	NR	NR	329	10	4
			Ustekinumab	313	217	19	179	NR	NR	168	2	1
			Placebo	315	19	1	13	NR	NR	152	3	0

Papp	Brodalumab	12	210 mg	40	33	25	32	NR	NR	33	1	0
			140 mg	39	30	15	33	NR	NR	27	0	0
			Placebo	38	0	0	1	NR	NR	23	1	0

Nakagawa	Brodalumab	12	210 mg	37	35	22	35	NR	NR	27	1	0
			140 mg	37	29	13	29	NR	NR	21	0	0
			Placebo	38	3	0	2	NR	NR	17	1	1

Rich	Secukinumab	12	150 mg	133	72	NR	NR	49	NR	89	6	3
			Placebo	67	1	NR	NR	1	NR	47	1	1

Paul (JUNCTURE)	Secukinumab	12	300 mg	60	52	16	NR	44	NR	42	1	0
			150 mg	61	44	10	NR	33	NR	39	3	0
			Placebo	61	2	0	NR	0	NR	33	1	1

Langley (ERASURE)	Secukinumab	12	300 mg	245	200	70	NR	160	NR	135	4	5
			150 mg	245	174	31	NR	125	NR	148	4	4
			Placebo	248	11	2	NR	6	NR	116	4	4

Langley (FIXTURE)	Secukinumab	12	300 mg	327	249	78	NR	202	NR	181	4	4
			150 mg	327	219	47	NR	167	NR	191	7	2
			Placebo	326	16	0	NR	9	NR	163	6	3

Blauvelt (FEATURE)	Secukinumab	12	300 mg	59	46	25	NR	41	NR	30	3	1
			150 mg	59	41	5	NR	31	NR	34	0	0
			Placebo	59	0	0	NR	0	NR	28	1	1

Gordon (UNCOVER-1)	Ixekizumab	12	80 mg Q4W	432	357	145	330	NR	NR	264	12	10
			80 mg Q2W	433	386	153	354	NR	NR	257	6	10
			Placebo	431	17	2	14	NR	NR	210	5	6

Griffiths (UNCOVER-2)	Ixekizumab	12	80 mg Q4W	347	269	107	253	NR	NR	204	8	5
			80 mg Q2W	351	315	142	292	NR	NR	216	5	4
			Placebo	168	4	1	4	NR	NR	89	2	1

Griffiths (UNCOVER-3)	Ixekizumab	12	80 mg Q4W	386	325	135	291	NR	NR	215	6	9
			80 mg Q2W	385	336	145	310	NR	NR	205	9	8
			Placebo	193	14	0	13	NR	NR	70	5	2

IGARASHI	Ustekinumab	12	45 mg	64	38	NR	NR	NR	37	42	0	0
			90 mg	62	42	NR	NR	NR	43	37	3	4
			Placebo	32	2	NR	NR	NR	3	21	2	2

Leonardi (PHOENIX 1)	Ustekinumab	12	45 mg	255	171	32	NR	NR	154	147	2	1
			90 mg	256	170	28	NR	NR	158	131	4	4
			Placebo	255	8	0	NR	NR	10	123	2	6

Papp (PHOENIX 2)	Ustekinumab	12	45 mg	409	273	74	NR	NR	278	217	8	1
			90 mg	411	311	75	NR	NR	302	197	5	6
			Placebo	410	15	0	NR	NR	20	204	8	8

Tsai (PEARL)	Ustekinumab	12	45 mg	61	41	5	NR	NR	43	40	0	0
			Placebo	60	3	0	NR	NR	5	42	2	3

Zhu(LOTUS)	Ustekinumab	12	45 mg	160	132	38	NR	NR	126	68	1	3
			Placebo	162	18	1	NR	NR	24	62	1	2

Krueger	Ustekinumab	12	45 mg	64	43	10	NR	NR	46	49	2	1
			90 mg	64	52	13	NR	NR	53	42	3	1
			Placebo	64	1	0	NR	NR	0	48	1	2

Blauvelt (VOYAGE 1)	Guselkumab	16	100 mg	329	300	123	NR	280	NR	170	8	4
			Placebo	174	10	1	NR	12	NR	86	3	2

Reich (VOYAGE 2)	Guselkumab	16	100 mg	496	328	169	NR	417	NR	235	8	7
			Placebo	248	20	2	NR	21	NR	111	3	2

Gordon	Guselkumab	16	50 mg	42	34	8	NR	NR	33	21	3	NA
			100 mg	42	33	14	NR	NR	36	19	0	5
			Placebo	42	2	0	NR	NR	3	22	1	3

OHTSUKI	Guselkumab	16	50 mg	65	58	21	NR	60	NR	30	1	1
			100 mg	63	53	17	NR	56	NR	29	1	0
			Placebo	64	4	0	NR	5	NR	36	2	6

Papp	Tildrakizumab	12	100 mg	89	54	NA	NR	NR	55	58	1	1
			200 mg	86	62	NA	NR	NR	64	54	2	1
			Placebo	46	2	NA	NR	NR	1	31	0	1

Reich (reSURFACE 1)	Tildrakizumab	12	100 mg	309	197	43	NR	NR	179	146	5	0
			200 mg	308	192	43	NR	NR	182	130	8	5
			Placebo	155	9	2	NR	NR	11	74	1	1

Reich (reSURFACE 2)	Tildrakizumab	12	100 mg	307	188	38	NR	NR	168	136	4	3
			200 mg	314	206	37	NR	NR	186	155	6	3
			Placebo	156	9	0	NR	NR	7	86	4	2

Gordon (UltIMMa-1)	Risankizumab	16	150 mg	304	264	109	267	NR	NR	151	7	2
			Ustekinumab	100	70	12	63	NR	NR	50	8	2
			Placebo	102	10	0	8	NR	NR	52	3	4

Gordon (UltIMMa-2)	Risankizumab	16	150 mg	294	261	149	246	NR	NR	134	6	1
			Ustekinumab	99	69	24	62	NR	NR	53	3	0
			Placebo	98	8	2	5	NR	NR	45	1	1

PASI 75: the percentages of patients with a 75% improvement from baseline in the PASI score; PASI 100: the percentages of patients with a 100% improvement from baseline in the PASI score; sPGA 0/1: static physician's global assessment score of 0 or 1; IGA 0/1: a response of 0 or 1 on the modified investigator's global assessment; PGA 0/1: physician's global assessment score of 0 or 1; AEs: adverse events; sAEs: serious adverse events; Q2W: every 2 weeks; Q4W: every 4 weeks; NR: not reported; NA: not available.

**Table 3:** Ranking for all the outcomes of the interventions included at 12 or 16 weeks in the network meta-analysis.

Interventions	PASI 75			PASI 100			sPGA 0/1 or IGA 0/1 or PGA 0/1			AEs			sAEs			Discontinuations due to AEs		
	SUCRA (%)	PrBest	MeanRank	SUCRA (%)	PrBest	MeanRank	SUCRA (%)	PrBest	MeanRank	SUCRA (%)	PrHighest risk	MeanRank	SUCRA (%)	PrHighest risk	MeanRank	SUCRA (%)	PrHighest risk	MeanRank
Brodalumab 140 mg	22.8	0.0	11.0	63.4	0.5	5.8	32.0	0.0	9.8	38.1	0.0	9.0	38.4	0.1	9.0	54.2	0.6	7.0
Brodalumab 210 mg	62.5	0.3	5.9	85.0	28.4	2.9	65.4	0.1	5.5	23.7	0.0	10.9	63.9	3.8	5.7	63.0	0.9	5.8
Guselkumab 100 mg	28.5	0.0	10.3	61.4	6.0	6.0	31.4	0.0	9.9	63.4	1.3	5.8	49.4	3.3	7.6	32.1	0.2	9.8
Guselkumab 50 mg	38.7	0.4	9.0	55.9	4.3	6.7	33.4	0.0	9.7	76.2	30.1	4.1	25.9	3.5	10.6	84.6	36.7	3.0
Ixekizumab 80 mg Q2W	93.0	53.4	1.9	83.3	32.7	3.2	86.5	13.3	2.8	7.5	0.0	13.0	50.8	3.1	7.4	14.8	0.0	12.1
Ixekizumab 80 mg Q4W	81.5	0.5	3.4	76.8	12.9	4.0	75.7	0.0	4.2	4.5	0.0	13.4	27.5	0.5	10.4	10.7	0.0	12.6
Placebo	0.0	0.0	14.0	0.0	0.0	14.0	0.0	0.0	14.0	67.3	0.0	5.3	59.4	0.1	6.3	39.7	0.0	8.8
Risankizumab 150 mg	62.3	0.4	5.9	71.3	6.4	4.7	66.7	0.4	5.3	67.6	5.3	5.2	92.8	61.5	1.9	92.6	53.2	2.0
Secukinumab 150 mg	73.5	0.0	4.4	31	0.1	10.0	85.7	0.0	2.9	22.7	0.0	11.0	30.7	0.4	10.0	43.5	0.2	8.4
Secukinumab 300 mg	89.9	43.1	2.3	62.4	8.3	5.9	98.1	86.0	1.3	33.9	0.0	9.6	35.6	0.9	9.4	42.2	0.1	8.5
Tildrakizumab 100 mg	33.2	0.2	9.7	21.9	0.4	11.2	22.3	0.0	11.1	88.8	27.9	2.5	70.8	18.9	4.8	58.6	4.5	6.4
Tildrakizumab 200 mg	42.2	1.7	8.5	20.0	0.0	11.4	34.7	0.2	9.5	90.2	31.3	2.3	41.1	1.2	8.7	35.4	0.4	9.4
Ustekinumab 45 mg	27.4	0.0	10.4	33.1	0.0	9.7	25.4	0.0	10.7	41.0	0.0	8.7	61.1	0.6	6.1	79.0	3.0	3.7
Ustekinumab 90 mg	44.5	0.0	8.2	34.5	0.0	9.5	42.8	0.0	8.4	75.2	4.1	4.2	52.5	2.1	7.2	49.7	0.2	7.5

SUCRA: surface under the cumulative ranking; PrBest: probability of best treatment; PrHighest risk: probability of the highest risk of AEs; PASI 75: the percentages of patients with a 75% improvement from baseline in the PASI score; PASI 100: the percentages of patients with a 100% improvement from baseline in the PASI score; sPGA 0/1: static physician's global assessment score 0 or 1; IGA 0/1: a response of 0 or 1 on the modified investigator's global assessment; PGA 0/1: physician's global assessment score of 0 or 1; AEs: adverse events; sAEs: serious adverse events; Q2W: every 2 weeks; Q4W: every 4 weeks.

## Supplementary Materials

Supplementary Materials Supplementary Table 1: search strategy. Supplementary Figure 1: interval plot between interventions versus placebo of achieving PASI 75 at 12 or 16 weeks in network meta-analysis. Supplementary Figure 2: interval plot between interventions versus placebo of achieving PASI 100 at 12 or 16 weeks in network meta-analysis. Supplementary Figure 3: interval plot between interventions versus placebo of achieving sPGA 0/1 or IGA 0/1 or PGA 0/1 at 12 or 16 weeks in network meta-analysis. Supplementary Figure 4: interval plot between interventions versus placebo of achieving one or more AEs at 12 or 16 weeks in network meta-analysis. Supplementary Figure 5: interval plot between interventions versus placebo of achieving one or more sAEs at 12 or 16 weeks in network meta-analysis. Supplementary Figure 6: interval plot between interventions versus placebo of discontinuations due to AEs at 12 or 16 weeks in network meta-analysis. Supplementary Figure 7: interval plot of sensitivity analyses by excluding the trials at the high risk of bias for achieving PASI 100 at 12 or 16 weeks. Supplementary Figure 8: interval plot of sensitivity analyses by excluding the trials at the high risk of bias for achieving sPGA 0/1 or IGA 0/1 or PGA 0/1 at 12 or 16 weeks.
