# Supplementary material for: Short-Term Efficacy and Safety of IL-17, IL-12/23, and IL-23 Inhibitors Brodalumab, Secukinumab, Ixekizumab, Ustekinumab, Guselkumab, Tildrakizumab, and Risankizumab for the Treatment of Moderate to Severe Plaque Psoriasis: A Systematic Review and Network Meta-Analysis of Randomized Controlled Trials
Source: J Immunol Res. 2019 Sep 10;2019:2546161. doi: 10.1155/2019/2546161 (PMC6754904; doi:10.1155/2019/2546161)
Supplement: Supplementary Materials — Supplementary Table 1: search strategy. Supplementary Figure 1: interval plot between interventions versus placebo of achieving PASI 75 at 12 or 16 weeks in network meta-analysis. Supplementary Figure 2: interval plot between interventions versus placebo of achieving PASI 100 at 12 or 16 weeks in network meta-analysis. Supplementary Figure 3: interval plot between interventions versus placebo of achieving sPGA 0/1 or IGA 0/1 or PGA 0/1 at 12 or 16 weeks in network meta-analysis. Supplementary Figure 4: interval plot between interventions versus placebo of achieving one or more AEs at 12 or 16 weeks in network meta-analysis. Supplementary Figure 5: interval plot between interventions versus placebo of achieving one or more sAEs at 12 or 16 weeks in network meta-analysis. Supplementary Figure 6: interval plot between interventions versus placebo of discontinuations due to AEs at 12 or 16 weeks in network meta-analysis. Supplementary Figure 7: interval plot of sensitivity analyses by excluding the trials at the high risk of bias for achieving PASI 100 at 12 or 16 weeks. Supplementary Figure 8: interval plot of sensitivity analyses by excluding the trials at the high risk of bias for achieving sPGA 0/1 or IGA 0/1 or PGA 0/1 at 12 or 16 weeks. [file 2546161.f1.docx]

**SUPPLEMENTARY TABLES, FIGURES AND APPENDICES**

**Supplementary Table 1. Search strategy**

| **Searched database** | | **Search strategy Quantities of documents** | | |
| --- | --- | --- | --- | --- |
| **Pubmed** | | #1 Psoriasis [Title/Abstract]  #2 Secukinumab [Title/Abstract]  #3 Cosentyx [Title/Abstract]  #4 AIN 457 [Title/Abstract]  #5 Brodalumab [Title/Abstract]  #6 Siliq [Title/Abstract]  #7 AMG-827 [Title/Abstract]  #8 lumicef [Title/Abstract]  #9 Ixekizumab [Title/Abstract]  #10 Taltz [Title/Abstract]  #11 LY2439821 [Title/Abstract]  #12 Ustekinumab [Title/Abstract]  #13 Stelara [Title/Abstract]  #14 CNTO 1275 [Title/Abstract]  #15 Tildrakizumab [Title/Abstract]  #16 SCH 900222 [Title/Abstract]  #17 MK-3222 [Title/Abstract]  #18 Risankizumab [Title/Abstract]  #19 BI 655066 [Title/Abstract]  #20 Guselkumab [Title/Abstract]  #21 #2 OR #3 OR #4 OR #5 OR #6 OR #7 OR #8 OR #9 OR #10 OR #11 OR #12 OR #13 OR #14 OR #15 OR #16 OR #17 OR #18 OR #19 OR #20  #22 #1 AND #21 | | 36017  504  14  4  165  6  8  3  247  8  7  1242  26  15  48  0  4  24  5  74  1821  1217 |
| **Embase** | | #1 ‘psoriasis'/exp OR psoriasis  #2 ‘Secukinumab '/exp OR Secukinumab  #3 ‘Cosentyx '/exp OR Cosentyx  #4 ‘AIN 457'/exp OR AIN 457  #5 ‘Brodalumab '/exp OR Brodalumab  #6 ‘Siliq '/exp OR Siliq  #7 ‘AMG-827'/exp OR AMG-827  #8 ‘lumicef '/exp OR lumicef  #9 ‘Ixekizumab '/exp OR Ixekizumab  #10 ‘Taltz '/exp OR Taltz  #11 ‘LY2439821 '/exp OR LY2439821  #12 ‘Ustekinumab '/exp OR Ustekinumab  #13 ‘Stelara '/exp OR Stelara  #14 ‘CNTO 1275'/exp OR CNTO 1275  #15 ‘Tildrakizumab '/exp OR Tildrakizumab  #16 ‘SCH 900222'/exp OR SCH 900222  #17 ‘MK-3222'/exp OR MK-3222  #18 ‘Risankizumab '/exp OR Risankizumab  #19 ‘BI 655066'/exp OR BI 655066  #20 ‘Guselkumab '/exp OR Guselkumab  #21 #2 OR #3 OR #4 OR #5 OR #6 OR #7 OR #8 OR #9 OR #10 OR #11 OR #12 OR #13 OR #14 OR #15 OR #16 OR #17 OR #18 OR #19 OR #20  #22 #1 AN D #21 | | 84688  2002  1949  2028  678  660  668  7  962  940  940  4773  4678  4672  165  25  41  115  120  266  6669  4827 |
| **Cochrane Central Register of Controlled Trials** | | #1 psoriasis  #2 Secukinumab  #3 Cosentyx  #4 AIN 457  #5 Brodalumab  #6 Siliq  #7 AMG-827  #8 lumicef  #9 Ixekizumab  #10 Taltz  #11 LY2439821  #12 Ustekinumab  #13 Stelara  #14 CNTO 1275  #15 Tildrakizumab  #16 SCH 900222  #17 MK-3222  #18 Risankizumab  #19 BI 655066  #20 Guselkumab  #21 #2 OR #3 OR #4 OR #5 OR #6 OR #7 OR #8 OR #9 OR #10 OR #11 OR #12 OR #13 OR #14 OR #15 OR #16 OR #17 OR #18 OR #19 OR #20  #22 #1 AND #21 | | 5509  464  3  0  74  0  23  0  211  2  24  408  8  6  18  22  6  34  21  56  1152  757 |
| **Total** |  | | 6801 | |

**
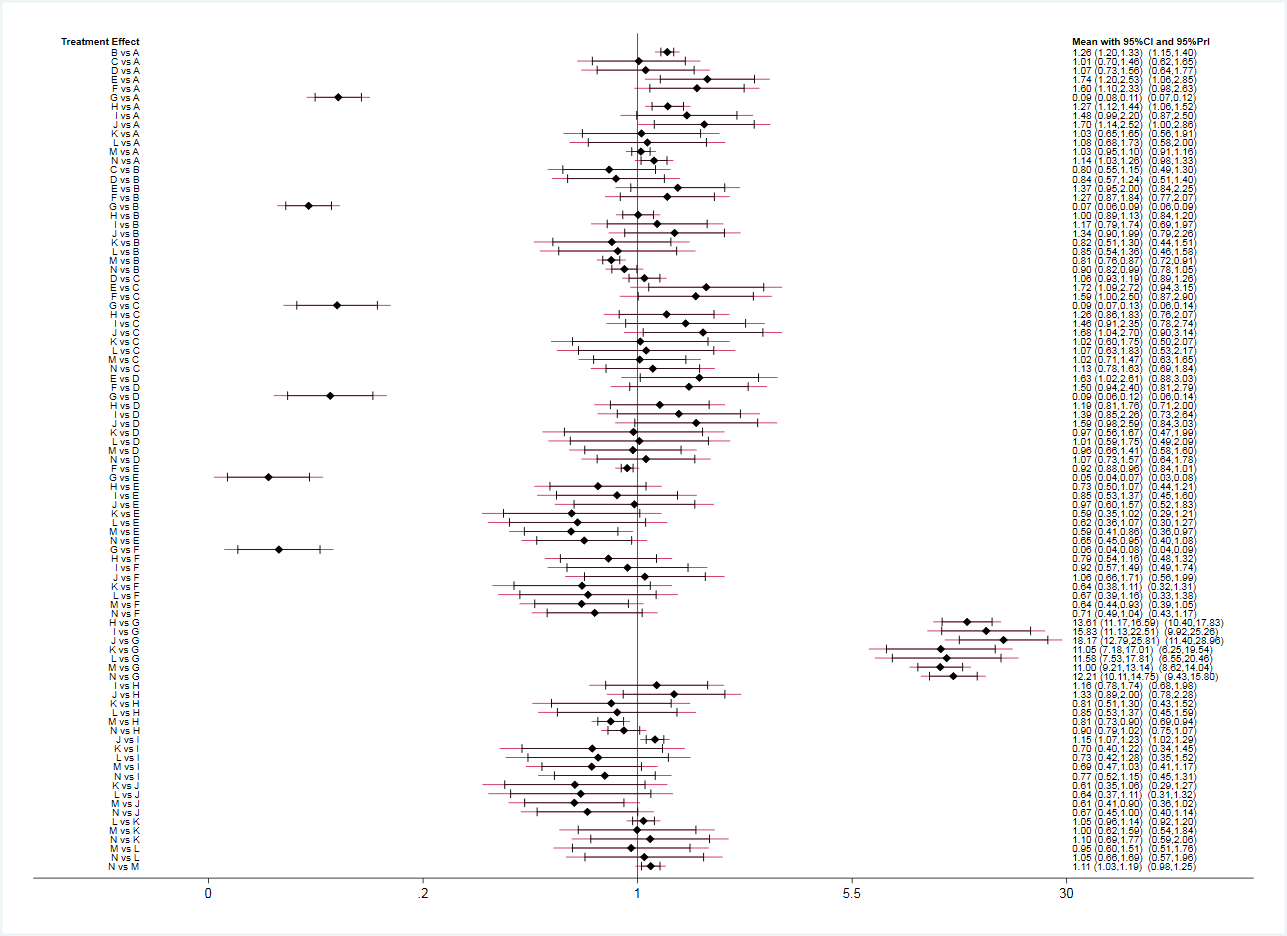
Supplementary Figure 1. Interval plot between interventions versus placebo of achieving PASI 75 at 12 or 16 weeks in network meta-analysis.**

**
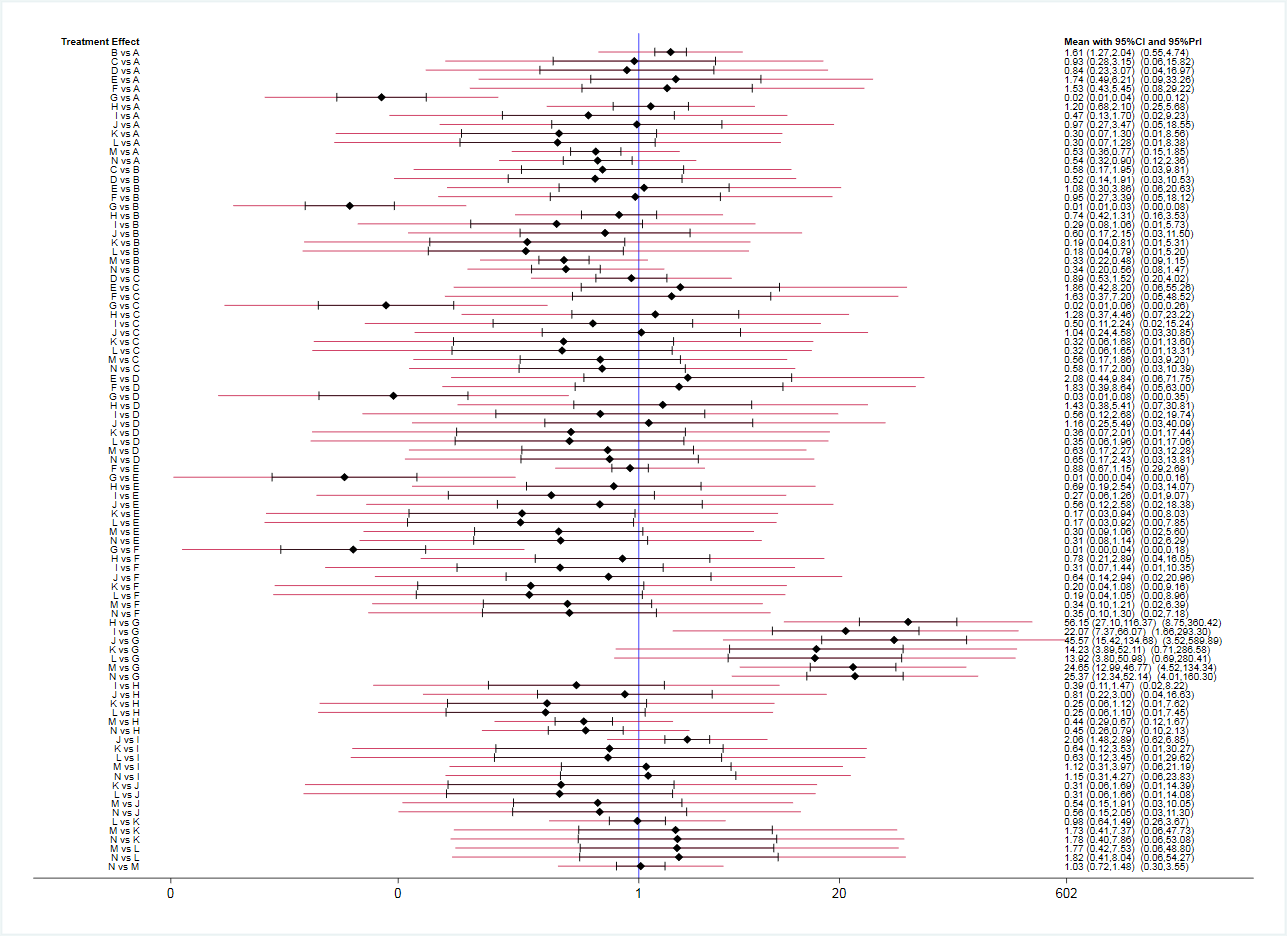
Supplementary Figure 2. Interval plot between interventions versus placebo of achieving PASI 100 at 12 or 16 weeks in network meta-analysis.**

**Supplementary Figure 3. Interval plot between interventions versus placebo of achieving sPGA0/1 or IGA0/1 or PGA0/1 at 12 or 16 weeks in network meta-analysis.**

**
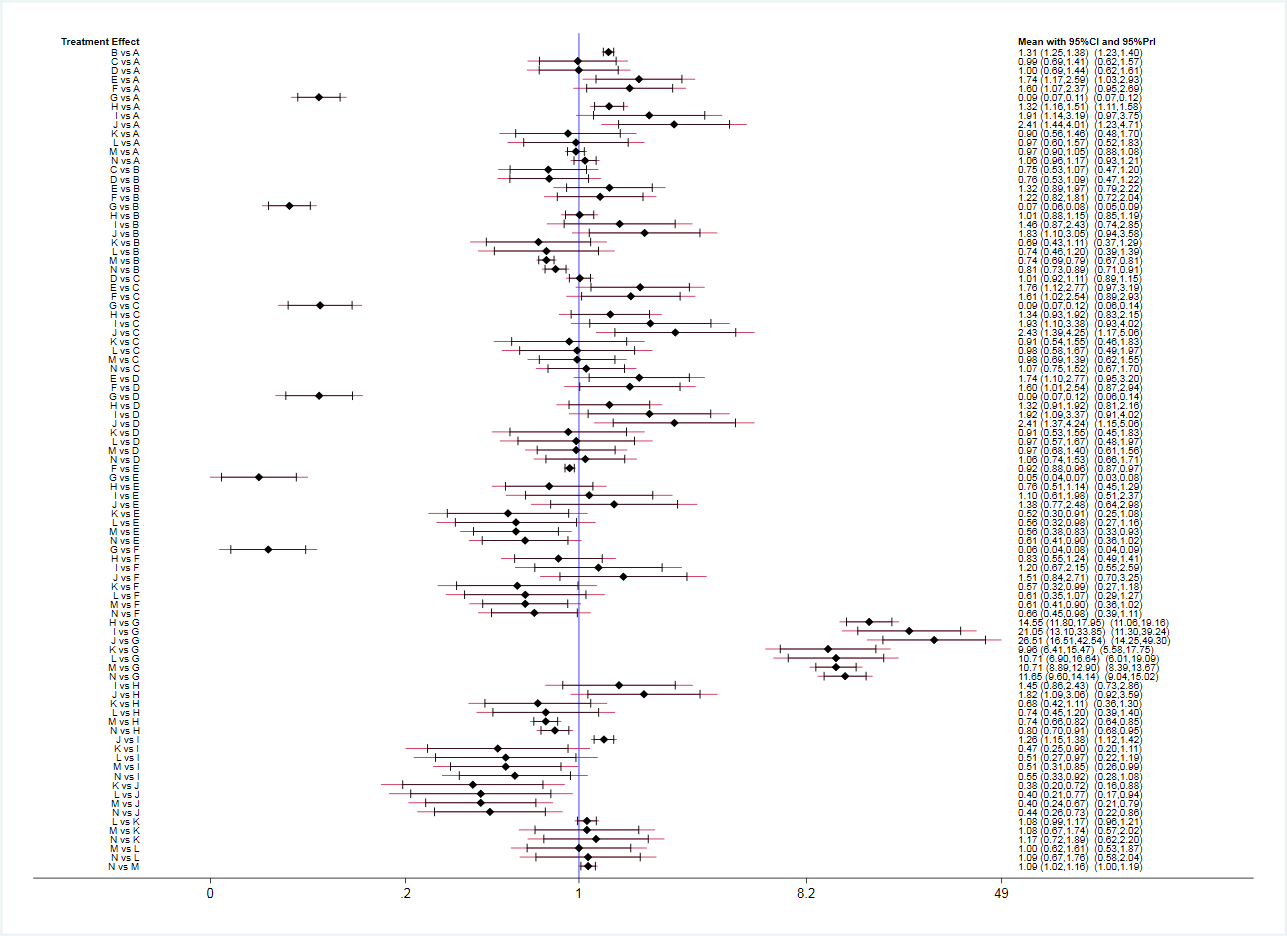
**

**Supplementary Figure 4. Interval plot between interventions versus placebo of achieving one or more AEs at 12 or 16 weeks in network meta-analysis.**

**
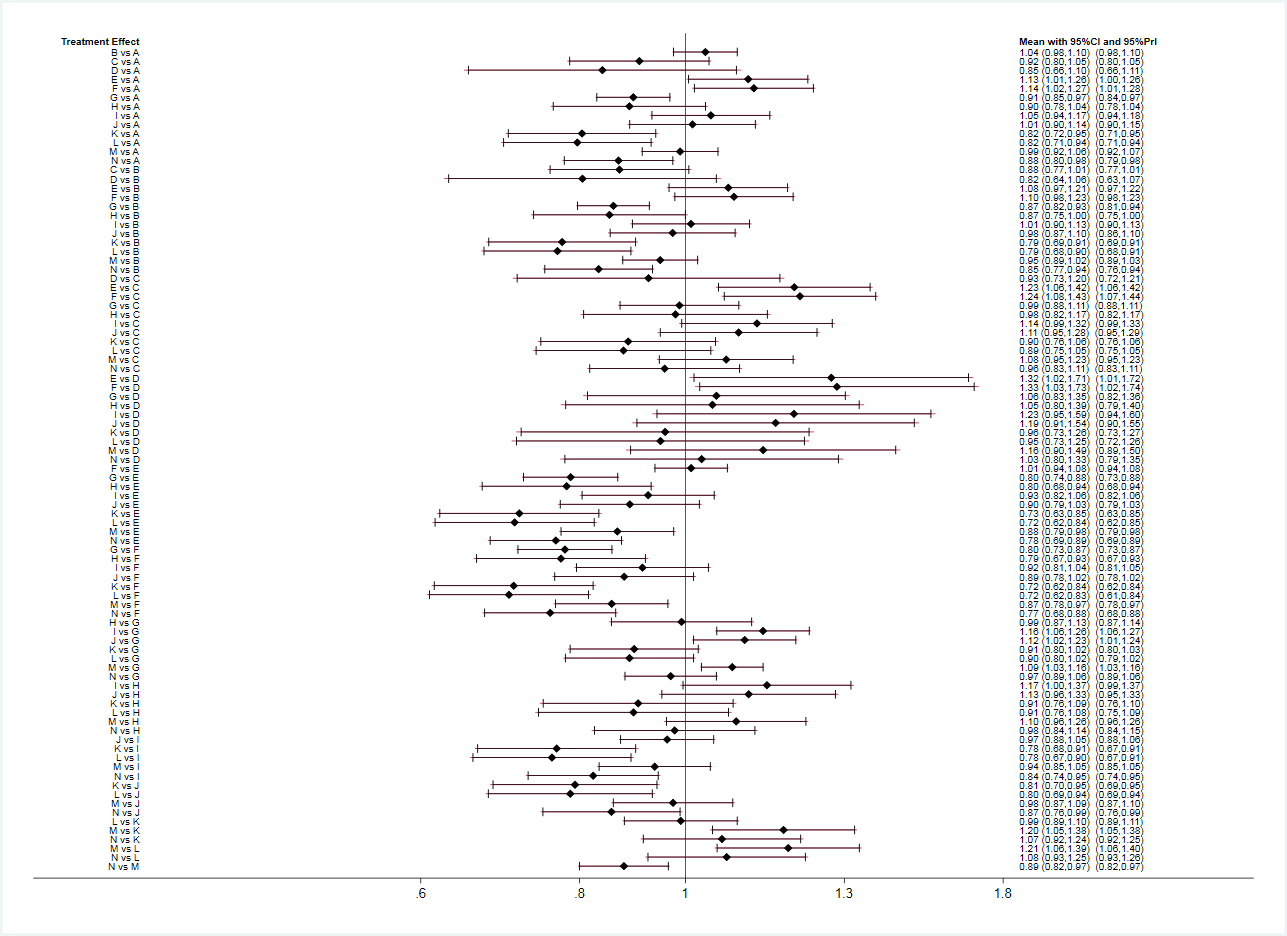
**

**Supplementary Figure 5. Interval plot between interventions versus placebo of achieving one or more sAEs at 12 or 16 weeks in network meta-analysis.**

**
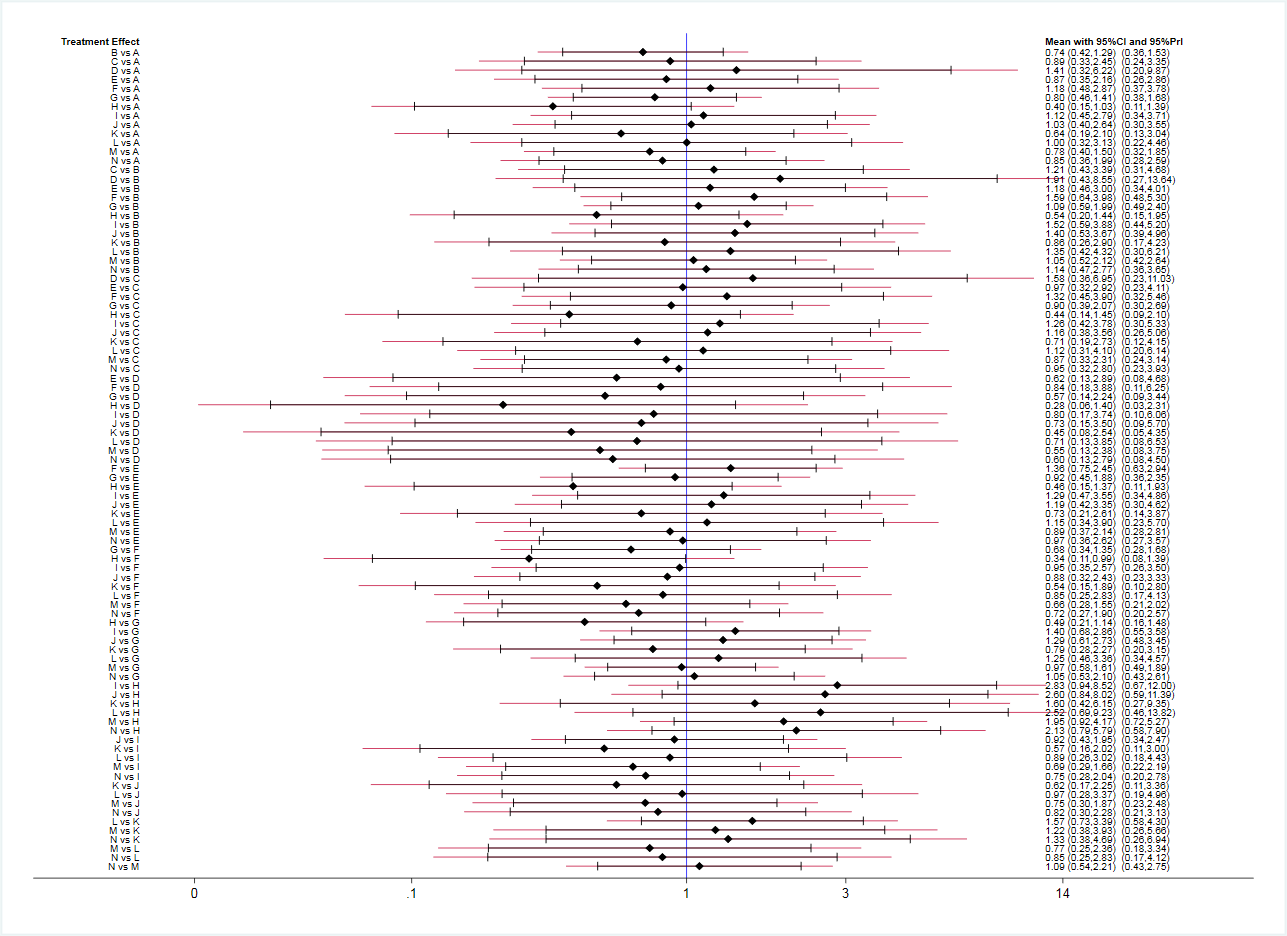
**

**Supplementary Figure 6. Interval plot between interventions versus placebo of discontinuations due to AEs at 12 or 16 weeks in network meta-analysis.**

**
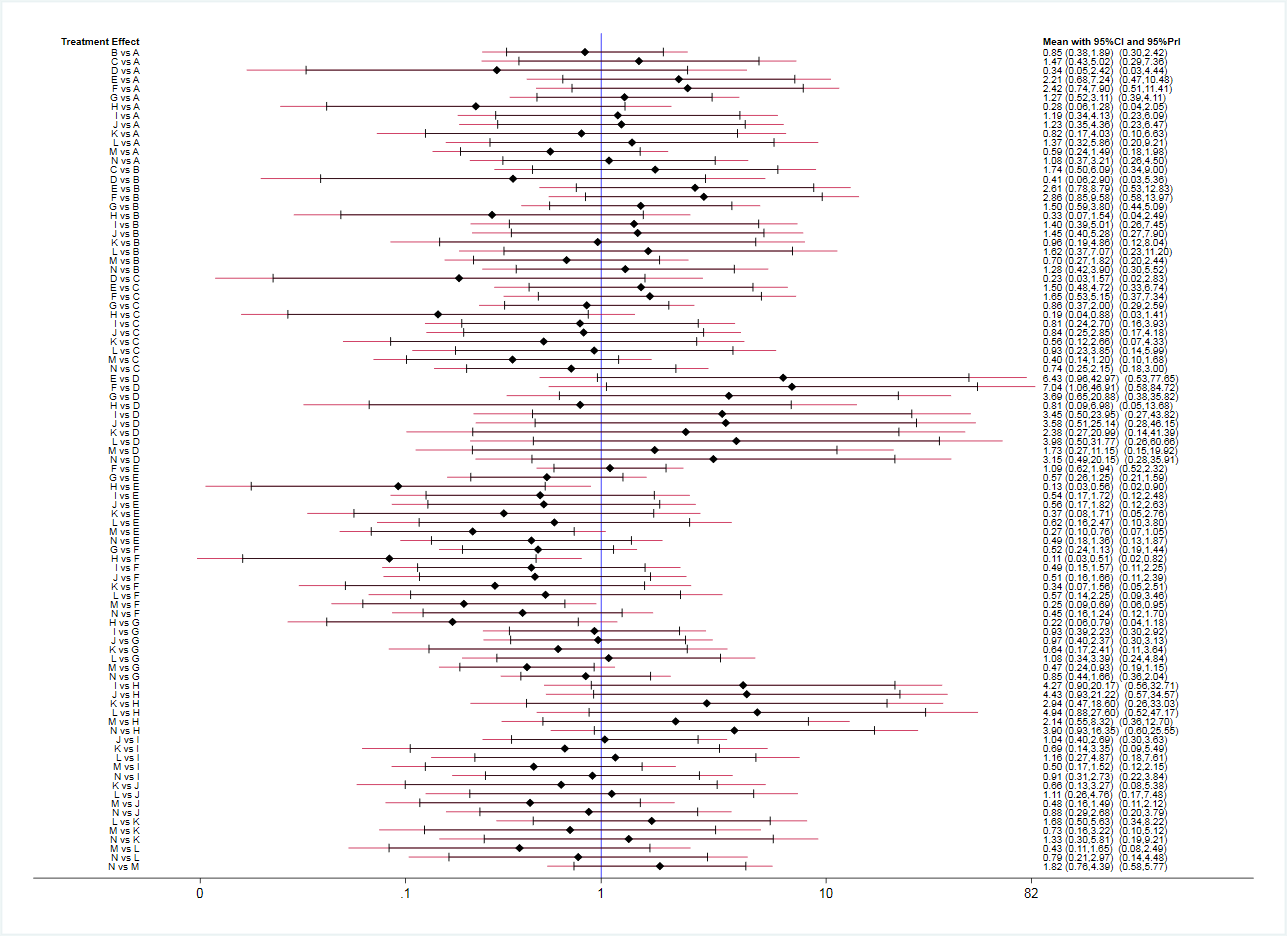
**

**Supplementary Figure 7. Interval plot of sensitivity analyses by excluding the trials at high risk of bias for achieving PASI 100 at 12 or 16 weeks.**

**
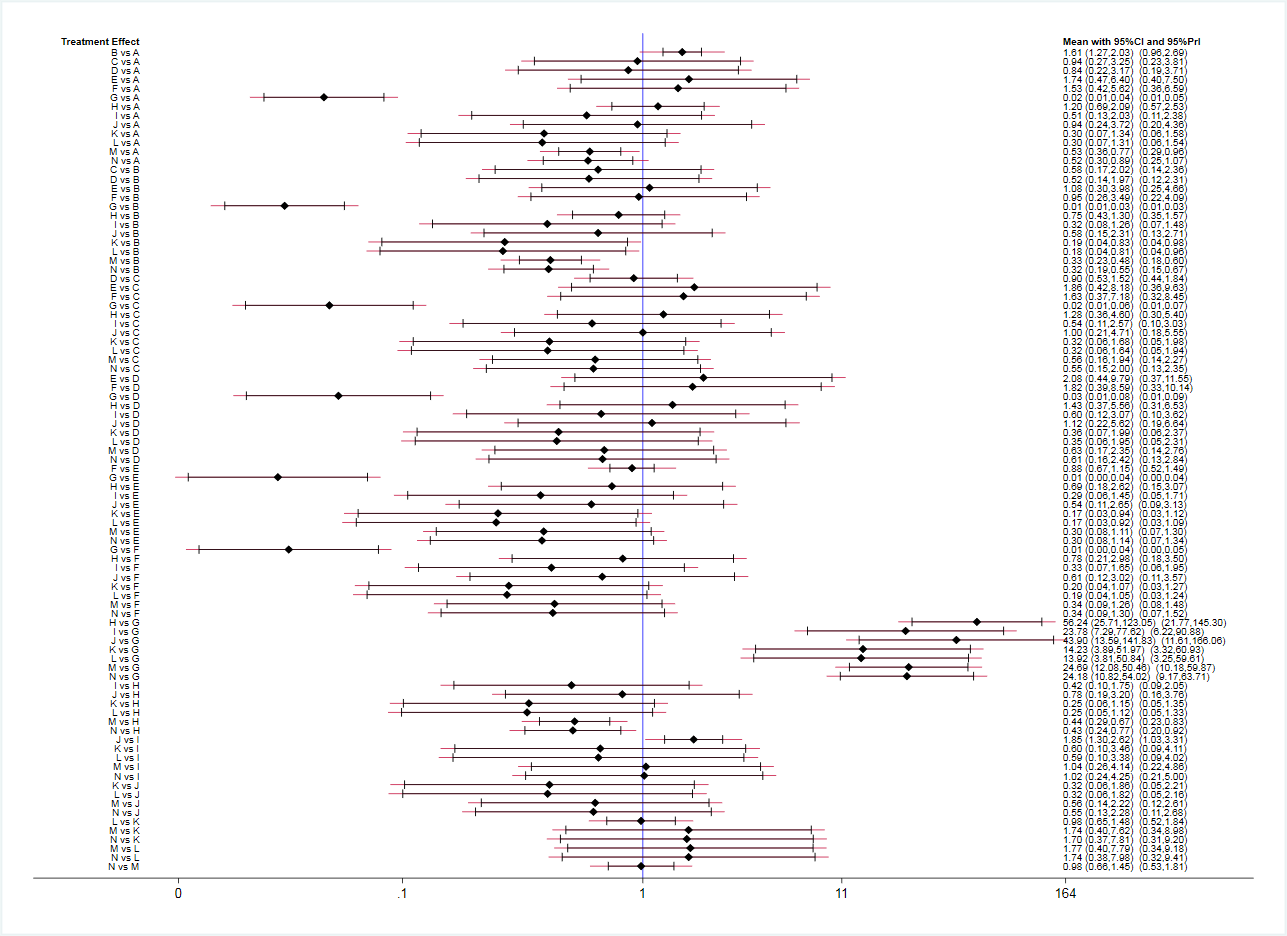
**

**Supplementary Figure 8. Interval plot of sensitivity analyses by excluding the trials at high risk of bias for achieving sPGA0/1 or IGA0/1 or PGA0/1 at 12 or 16 weeks.**

**
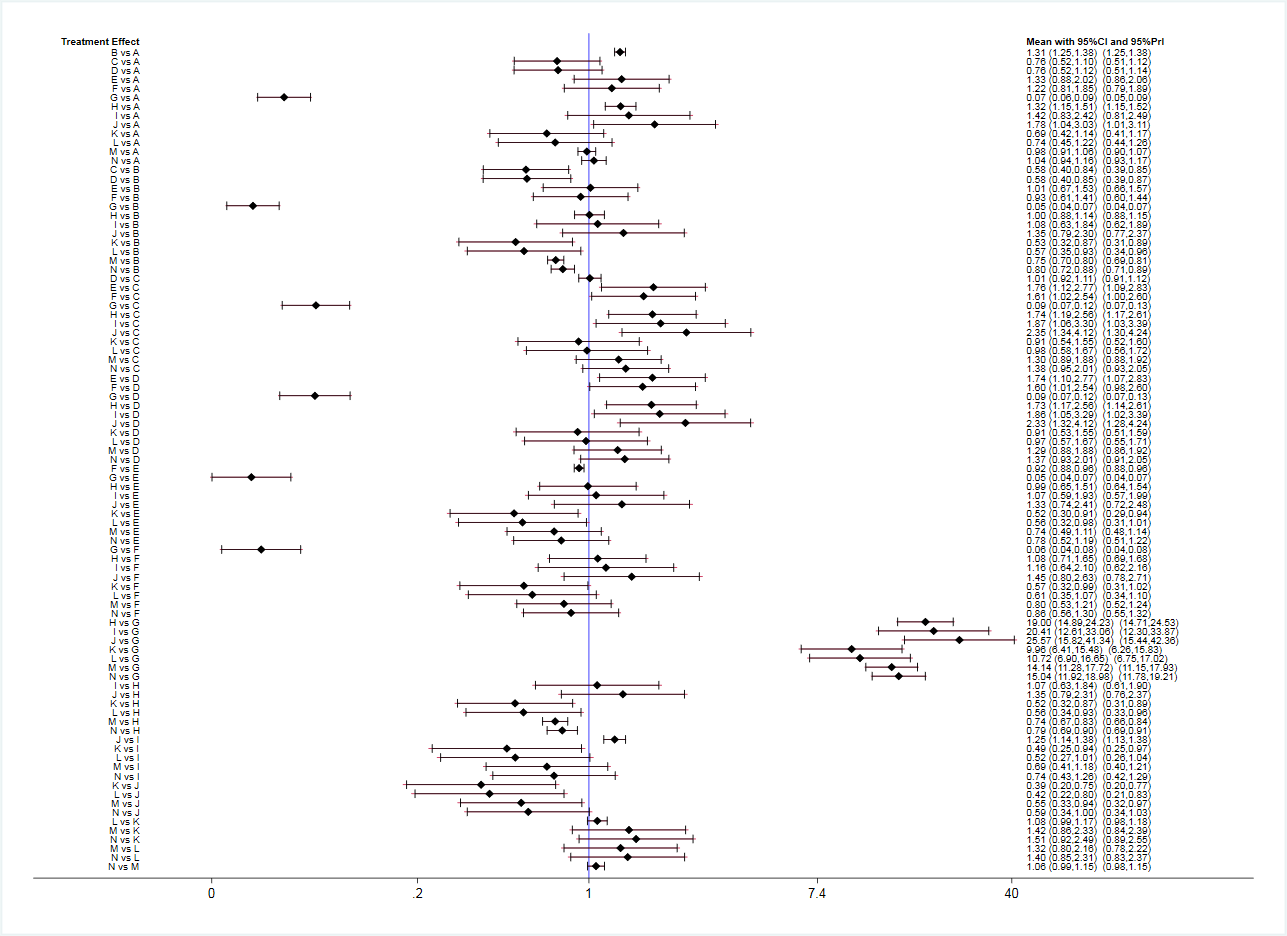
**

**Alphabetic reference: A, brodalumab 140 mg; B, brodalumab 210 mg; C, guselkumab 100 mg; D, guselkumab 50 mg; E, ixekizumab 80 mg Q2W; F, ixekizumab 80 mg Q4W; G, placebo; H, risankizumab 150 mg; I, secukinumab 150 mg; J, secukinumab 300 mg; K, tildrakizumab 100 mg; L, tildrakizumab 200 mg; M, ustekinumab 45 mg; and N, ustekinumab 90 mg.**
